# Four Theorems on the Psychometric Function

**DOI:** 10.1371/journal.pone.0074815

**Published:** 2013-10-04

**Authors:** Keith A. May, Joshua A. Solomon

**Affiliations:** Division of Optometry and Visual Science, City University London, London, United Kingdom; University of Ulm, Germany

## Abstract

In a 2-alternative forced-choice (2AFC) discrimination task, observers choose which of two stimuli has the higher value. The psychometric function for this task gives the probability of a correct response for a given stimulus difference, 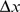. This paper proves four theorems about the psychometric function. Assuming the observer applies a transducer and adds noise, Theorem 1 derives a convenient general expression for the psychometric function. Discrimination data are often fitted with a Weibull function. Theorem 2 proves that the Weibull “slope” parameter, 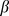, can be approximated by 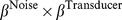, where 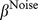 is the 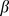 of the Weibull function that fits best to the cumulative noise distribution, and 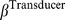 depends on the transducer. We derive general expressions for 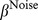 and 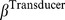, from which we derive expressions for specific cases. One case that follows naturally from our general analysis is Pelli's finding that, when 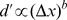, 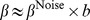. We also consider two limiting cases. Theorem 3 proves that, as sensitivity improves, 2AFC performance will usually approach that for a linear transducer, whatever the actual transducer; we show that this does not apply at signal levels where the transducer gradient is zero, which explains why it does not apply to contrast detection. Theorem 4 proves that, when the exponent of a power-function transducer approaches zero, 2AFC performance approaches that of a logarithmic transducer. We show that the power-function exponents of 0.4–0.5 fitted to suprathreshold contrast discrimination data are close enough to zero for the fitted psychometric function to be practically indistinguishable from that of a log transducer. Finally, Weibull 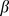 reflects the shape of the noise distribution, and we used our results to assess the recent claim that internal noise has higher kurtosis than a Gaussian. Our analysis of 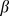 for contrast discrimination suggests that, if internal noise is stimulus-independent, it has *lower* kurtosis than a Gaussian.

## Introduction

On each trial of a 2-alternative forced-choice (2AFC) discrimination task, observers are presented with two stimuli, one (often called the *pedestal*) with stimulus value 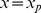, and one (the *target*) with value 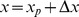, where 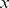 represents a value along some stimulus dimension, such as contrast, luminance, frequency, sound intensity, etc., and 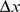 represents a (usually) positive increment in 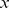. The observer has to say which stimulus contained the higher value, 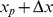. For this task, the function relating stimulus difference, 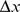, to the probability of a correct response, 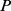, is called the *psychometric function*. The form of the psychometric function can reveal characteristics of the underlying mechanisms, helping to constrain the set of possible models. In this paper we present four theorems that help us to understand the properties of the psychometric function and clarify the relationship between the psychometric function and the underlying model.

In order to fit the psychometric function to data, we need a mathematical function whose parameters can be adjusted to fit the kind of data set usually obtained. A widely used function is the Weibull function, and two of our theorems relate specifically to this function. Letting 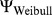 represent the Weibull function, and letting 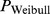 represent its output (i.e., the predicted proportion correct), the Weibull function is given by
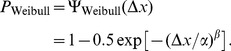
(1)

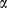
 is the “threshold” parameter, the stimulus increment that gives rise to a proportion correct of 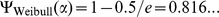. In what follows, we will frequently refer to this threshold performance level as 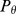, so this term should be read as the constant, 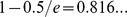. 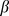 is often referred to as the “slope” parameter, because it is proportional to the gradient of the Weibull function at 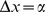 when 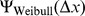 is plotted on a log abscissa.

**Figure 1:**
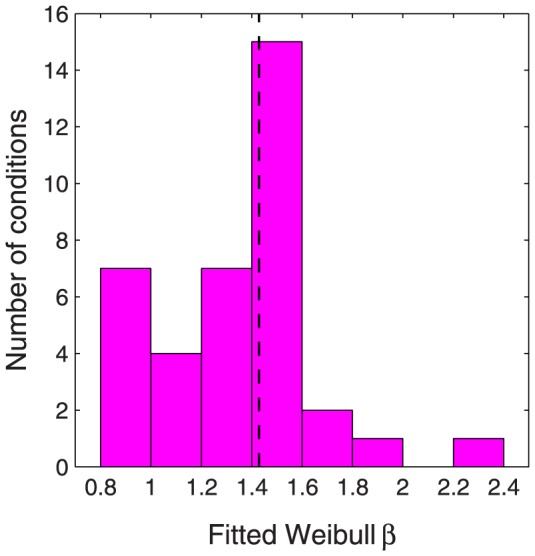
Distribution of fitted Weibull 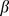 values in Table 1. The fitted 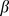 values from the suprathreshold (non-starred) conditions of Table 1 were dropped into bins with edges that stepped from 0.8 to 2.4 in jumps of 0.2 (the histogram thus excludes one outlier, the value 6.70 for Henning et al. 's [47] observer GBH at 8.37 cpd). For this histogram, we used the 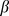 values that had been fitted using a nonzero lapse rate parameter where available, as this is more likely to reflect the true 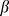. The median of this hybrid population (some including a lapse rate parameter, some not) was 1.43 (indicated by the vertical dashed line).

In 2AFC visual contrast discrimination experiments where the contrasts of both stimuli are at least as high as the detection threshold, 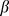 usually falls between 1 and 2, with a median of around 1.4 (see Table 1 and Figure 1). As the pedestal contrast approaches zero (making it a 2AFC contrast detection task), 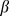 increases to a value of around 3 [1]–[5].

**Table 1:** Fitted Weibull function parameters for 2AFC contrast discrimination.

			*λ* = 0			*λ* fitted			
Study	Condition/observer	Pedestal	*α*	*β*	*W*	*α*	*β*	*λ*	*W*
Bird et al. [34]	CMB	0.03	0.00735	1.12	0.245	0.00735	1.12	5×10^−13^	0.245
	CMB	0.3	0.0779	1.11	0.260	0.0692	1.21	0.0309	0.231
	GBH	0.03	0.00737	0.734	0.246	0.00643	0.832	0.0251	0.214
	GBH	0.3	0.0574	0.952	0.191	0.0541	0.993	0.0128	0.180
Foley & Legge [1]	JMF, 0.5 cpd	0.00400	0.00165	1.35	0.412	0.00165	1.35	3×10^−9^	0.412
	JMF, 2 cpd	0.00230	0.00111	1.56	0.484	0.00111	1.56	5×10^−12^	0.484
	JMF, 8 cpd	0.00300	0.00125	1.44	0.418	0.00123	1.46	0.0071	0.410
	GW, 0.5 cpd	0.00400	0.00134	1.50	0.335	0.00134	1.50	2×10^−12^	0.335
	GW, 2 cpd	0.00229	0.000923	1.58	0.404	0.000923	1.58	1×10^−12^	0.404
	GW, 8 cpd	0.00330	0.00117	1.40	0.353	0.000996	1.94	0.0544	0.301
Henning et al. [47]	CMB 2.09 cpd	0.15	0.0421	1.49	0.281	0.0421	1.49	1×10^−12^	0.281
	CMB 8.37 cpd	0.15	0.0461	1.81	0.307	0.0379	2.21	0.0796	0.253
	GBH 2.09 cpd	0.15	0.0363	1.49	0.242	0.0363	1.49	2×10^−12^	0.242
	GBH 8.37 cpd	0.15	0.0401	1.21	0.267	0.0244	6.70	0.0645	0.163
Henning & Wichman [40]	GBH*	0*	0.0219*	4.26*	–*				
	GBH*	0.01*	0.0102*	13.1*	1.02*				
	GBH*	0.02*	0.00562*	1.67*	0.281*				
	GBH	0.04	0.00705	0.987	0.176				
	GBH	0.08	0.0156	1.16	0.195				
	GBH	0.16	0.0322	1.75	0.201				
	GBH	0.32	0.0773	1.45	0.241				
	NAL*	0*	0.00619*	4.84*	–*				
	NAL*	0.00141*	0.00492*	5.90*	3.48*				
	NAL*	0.00283*	0.00407*	2.28*	1.44*				
	NAL*	0.00566*	0.00224*	1.43*	0.395*				
	NAL	0.0113	0.00272	0.902	0.241				
	NAL	0.0226	0.00707	0.990	0.312				
	NAL	0.0453	0.0150	0.943	0.331				
	NAL	0.0905	0.0233	1.28	0.257				
	NAL	0.181	0.0424	1.59	0.234				
	NAL	0.362	0.0658	1.33	0.182				
	TCC*	0*	0.00838*	6.38*	–*				
	TCC*	0.005*	0.00443*	2.14*	0.886*				
	TCC	0.01	0.00339	0.912	0.339				
	TCC	0.016	0.00787	1.17	0.492				
	TCC	0.032	0.0126	1.52	0.393				
	TCC	0.08	0.0301	1.64	0.377				
	TCC	0.16	0.0381	1.27	0.238				
	TCC	0.32	0.0686	1.10	0.214				
Meese et al. [4]	Pedestal −∞ dB*	0*	0.00855*	3.32*	–*				
	Pedestal −10 dB*	0.00316*	0.00557*	2.44*	1.76*				
	Pedestal −5 dB*	0.00562*	0.00346*	1.47*	0.615*				
	Pedestal 0 dB	0.01	0.00340	1.47	0.340				
	Pedestal 5 dB	0.0178	0.00654	1.48	0.368				
	Pedestal 10 dB	0.0316	0.0110	1.40	0.348				
	Pedestal 15 dB	0.0562	0.0176	1.58	0.313				
	Pedestal 20 dB	0.1	0.0233	1.47	0.233				
	Pedestal 25 dB	0.178	0.0339	1.47	0.191				
	Pedestal 30 dB	0.316	0.0536	1.36	0.170				
Nachmias & Sansbury [6]	CS	0.0079	0.00387	1.27	0.489				
Mean of suprathreshold (non-starred) conditions				1.32	0.298		1.82		0.297
Median of suprathreshold conditions				1.38	0.274		1.49		0.267

**Figure 2:**
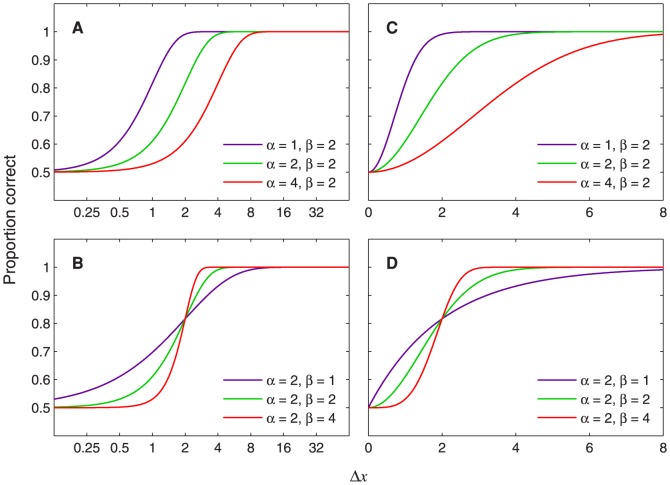
Effect of varying Weibull 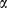 and 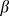 on log and linear abscissas. (A) Varying 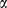 on a log abscissa: The curve shifts horizontally. (B) Varying 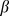 on a log abscissa: The curve is linearly stretched or compressed horizontally. (C) Varying 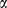 on a linear abscissa: The curve undergoes a linear horizontal stretch *and* a change of threshold. (D) Varying 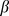 on a linear abscissa: The shape changes in a way that cannot be described as a linear scaling.

This table shows Weibull parameters fitted to 2AFC contrast discrimination data from six studies. The data from Meese et al. [4] are for their Binocular condition (plotted as squares in their Figure 5); these data were kindly provided by Tim Meese. For the other five papers, we read off the data points from digital scans of the figures (Bird et al. [34], Figure 1; Foley and Legge [1], Figure 1; Henning et al. [47], Figure 4 (sine wave stimuli only); Henning & Wichmann [40], Figure 4; Nachmias & Sansbury [6], Figure 2). In most cases, these figures plotted the proportion correct, 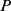, for several different contrast differences, 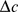, and we fitted the Weibull function using a maximum-likelihood method; specifically, we fitted the Weibull function by maximizing the expression 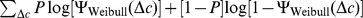, where 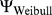 is the Weibull function whose parameters were being fitted. In Henning & Wichmann's [40] paper, the figures plotted the 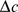 values corresponding to 60%, 75%, and 90% correct on the *fitted psychometric functions*, so we had to fit Weibull functions to points sampled from Henning & Wichmann's own fitted psychometric functions, rather than to the raw data. Where possible, we fitted both the lapse-free Weibull function of Equation (1), and the Weibull function of Equation (2), which includes a fitted lapse rate parameter, 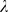. Parameters for the former fit appear under the heading “
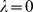
”, and those for the latter appear under the heading “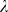 fitted”. In many cases, the data did not sufficiently constrain 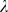 because there were no data points on the saturating portion of the psychometric function; in addition, Meese et al.'s Weibull fits did not include a lapse rate parameter. The Weber fraction, 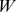, is given by 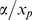, where 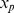 is the pedestal value. The means and medians at the bottom of the table are calculated from those studies for which the pedestal level exceeds the detection threshold, so that both stimuli were clearly visible. The cases where the pedestal is below detection threshold are starred in the table, and these were excluded from the means and medians.

When 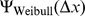 is plotted on a log abscissa, changing the value of 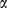 shifts the function horizontally, but otherwise leaves it unchanged (Figure 2A), and changing the value of 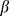 linearly stretches or compresses the function horizontally, leading to a change of slope (Figure 2B). On this log abscissa, the Weibull function always has the same basic shape, up to a linear horizontal scaling. When 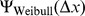 is plotted on a linear abscissa, changing the value of 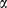 linearly stretches or compresses the function horizontally as well as changing the threshold (Figure 2C), while changing the value of 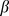 changes the shape of the function in a way that cannot be described as a linear horizontal scaling (Figure 2D).

Since 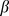 is proportional to the slope of the Weibull function on a log abscissa, the low value of 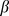 for contrast discrimination (compared with detection) often leads to the psychometric function for discrimination being described as “shallow”, and that for detection as “steep”. However, psychometric functions for contrast discrimination can be *steeper* than for detection when plotted on a *linear* contrast abscissa (e.g., Nachmias & Sansbury [6], Figure 2; Foley & Legge [1], Figure 1). We must therefore be vigilant not to be misled by the common practice of referring to 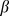 as the “slope” parameter. 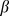 does control the slope of the Weibull function on a log abscissa, and this fact plays a key role in the proof of Theorem 2 of this paper, but the psychometric function is often plotted on a linear abscissa, and, in this case, 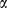 and 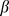 both affect the slope (Figures 2C and 2D); on a linear abscissa, 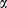 additionally controls the threshold and 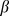 additionally controls the overall shape of the psychometric function. Thus, when considering a linear abscissa, it would be more appropriate to describe 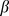 as the “shape” parameter, rather than the “slope” parameter.

The Weibull function defined in Equation (1) asymptotes to perfect performance (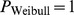). This is rarely achieved by human observers due to lapses of concentration, etc., and this can lead to a dramatic underestimation of 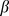 if the observer makes just one lapse on an easy trial [7]. Because of this problem, many researchers use a version of the Weibull function that includes a “lapse rate” parameter, 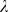:
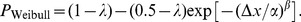
(2)This function asymptotes to 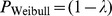, and reduces to Equation (1) in the case of 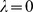. The psychometric function described by Equation (2) would result if the observer performed according to Equation (1) on a proportion 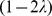 of trials, and guessed randomly on the remaining trials.

The Weibull function was originally proposed by Weibull [8] as a useful, general-purpose statistical distribution. Its widespread use as a psychometric function can be traced back to Quick [9], who was apparently unaware of Weibull's prior work. Quick proposed this function because, given certain assumptions, the Weibull function makes it easy to calculate how detection performance will be affected by adding extra stimulus components or increasing the size or duration of the stimulus, an approach that has become known as *probability summation* [10]–[13]. Quick focused on yes/no detection tasks, where the observer has to make a binary decision about a single stimulus (as opposed to the 2AFC tasks that we consider in this paper, in which the observer makes a binary decision about a pair of stimuli), but a similar analysis can be applied to 2AFC tasks [2].

Most treatments of probability summation with the Weibull function invoke the “high threshold assumption” that a zero-contrast stimulus never elicits a response in the detection mechanism, so detection errors are always unlucky guesses. This assumption makes a number of predictions that have turned out to be false [2], [14]–[16]. Furthermore, the convenient mathematics of probability summation with the Weibull function only applies to *detection*. For suprathreshold discrimination, where both stimuli are easily detectable, these computational benefits do not apply. Despite this, many researchers have continued to use the Weibull function to fit data from both detection and discrimination experiments for three perfectly valid reasons: it is well-known, fits well to the data, and is built into QUEST [17], probably the most widely used adaptive psychophysical method.

Different models of visual processing will deliver different mathematical forms for the psychometric function. Therefore, because of the widespread practice of fitting a Weibull function to data, it is of interest to know what happens when we fit a Weibull function to a psychometric function that is not a Weibull. In Theorem 2 of this paper, we derive a general analytical expression that gives a very accurate approximation of 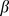 when the Weibull function is fitted to non-Weibull psychometric functions.

Although the usage of the Weibull function has its origin in outdated theoretical views, the Weibull function has very recently become more relevant again, due to the work of Neri [18]. He argues that the internal noise on the decision variable has a Laplace distribution, which, as we explain later in this Introduction, can lead to a psychometric function that has the form of a Weibull function with 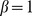.

First, we consider how the psychometric function might arise from the properties of the observer. In 2AFC discrimination experiments, the observer can be modelled using a transducer, followed by constant additive noise. The transducer converts the stimulus value, 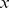, into some internal scalar signal value, 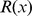. 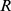 is called the *transducer function*. A noise sample from a stationary, stimulus-invariant distribution is then added to the internal signal, 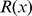, to give a noisy internal signal value. If the noise has zero mean, then 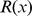 will be the mean internal signal for stimulus value 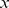. The observer compares the noisy internal signal values from the two stimuli, and chooses the stimulus that gave the higher value.

**Figure 3:**
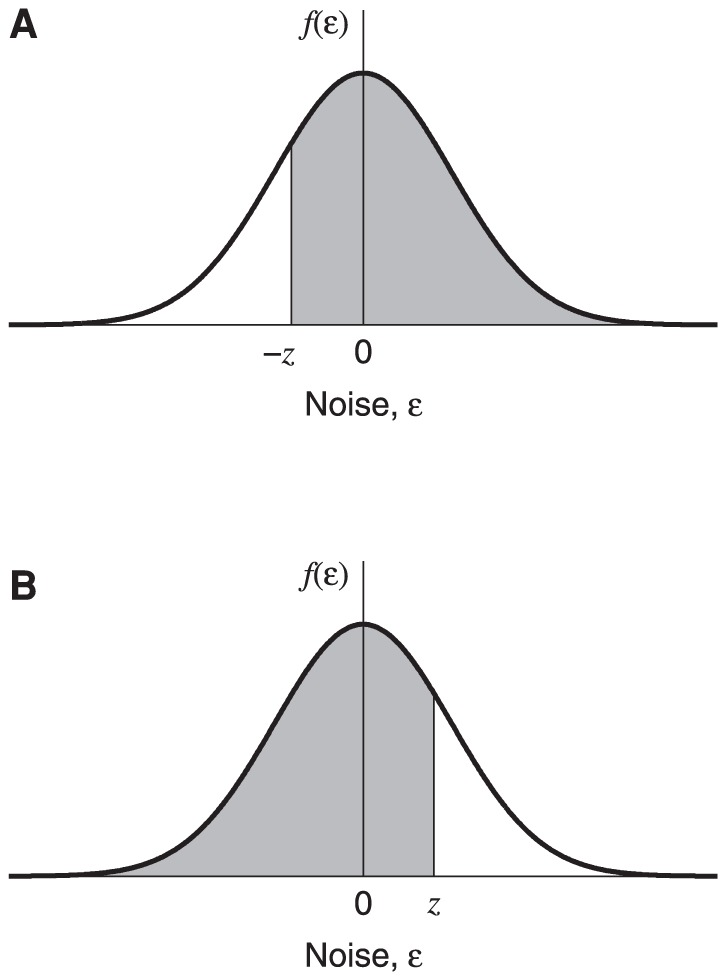
Graphical representation of the probability of a correct response. The shaded areas in A and B correspond to the integrals in Equations (4) and (5), respectively. The smooth curves trace out the PDF of the noise, 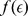, on the internal difference signal, 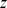. As explained in the text, 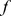 has to be even-symmetric, and this means that the two integrals in Equations (4) and (5) are equal. The shaded areas correspond to the probability of a correct response. The psychometric function (expressed as a function of 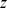) is the CDF of the noise, increasing from 0.5 to 1 as 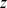 increases from 0 to 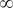.

From the experimenter's perspective, the observer behaves as if a sample of noise, 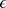, is added to the difference of mean signals, 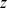, given by
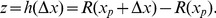
(3)The observer is correct when 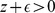, i.e. when 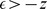. The probability, 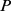, of this happening is given by
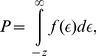
(4)where 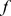 is the probability density function (PDF) of the noise, 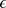. This integral corresponds to the shaded area in Figure 3A. 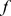 has to be even-symmetric, even if the noise added to the output of the transducer is not. This is because the noise sample on 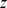 is equal to the noise sample on the target minus the noise sample on the nontarget. This is equivalent to swapping the sign of the nontarget noise sample and adding it to the target noise sample. The sign-reversed noise sample on the nontarget will have a PDF with mirror symmetry relative to the PDF of the noise sample on the target, so the sum of these two values will have an even-symmetric PDF. From the even symmetry of 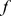 we have
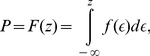
(5)where 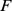 is the cumulative distribution function (CDF) of the observer's noise on the internal difference signal, and corresponds to the shaded area in Figure 3B. So the psychometric function for 2AFC discrimination, expressed as a function of 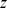, will trace out the positive half of the internal noise CDF, increasing from 0.5 to 1 as 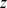 increases from 0 to 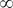.

**Figure 4:**
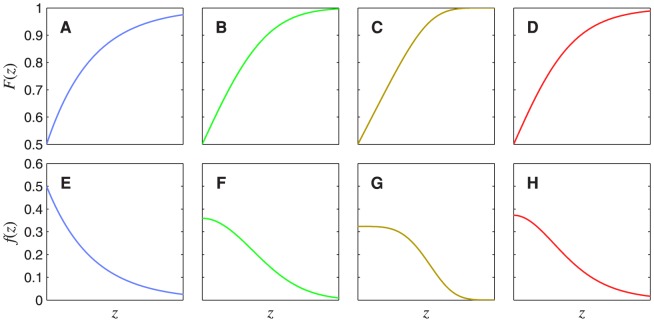
CDFs and PDFs of four different noise distributions. The top row shows noise CDFs, 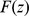, for (A) a Laplace distribution (generalized Gaussian with 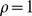), (B) a Gaussian distribution (generalized Gaussian with 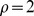), (C) a generalized Gaussian with 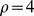, (D) a logistic distribution. Each panel in the bottom row shows the PDF, 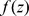, corresponding to the CDF above it. Only the positive halves of the distributions are shown (i.e. 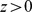). Note that the use of these colours for the different noise distributions is maintained in Figures 7, 8, 10, 11, 12, 15, and 16.

Figure 4 plots the CDFs and PDFs for several different forms of noise distribution (the mathematical definitions of these distributions will be given later). These CDFs (plotted as functions of 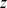) do not have a sigmoidal shape: The point of inflection is at zero on the abscissa. This is because the point of inflection corresponds to the peak of the derivative, and the derivative of these functions is the noise PDF, which peaks at 0 in each case.

In summary, 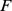 is the CDF of the internal noise, and takes an input of 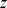 (Equation (5)); 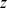 is the output of 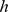, a function that is determined by the transducer and pedestal, and takes an input of 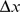 (Equation (3)). The composition of these two functions, 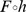, gives the observer's psychometric function when it is plotted as a function of 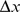. We use 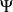 to represent this composition of functions:
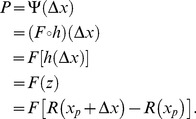
(6)If we fit the Weibull function, 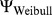, of Equation (1) to the psychometric function, 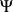, of Equation (6), then the Weibull slope parameter, 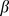, will be determined by both the noise CDF, 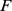, and the transducer, 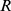. In Theorem 2, we show that, to a good approximation, 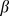 can be partitioned into a product of two factors, 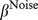 and 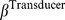. 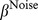 estimates the 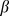 of the Weibull function that fits best to the noise CDF, while 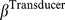 depends on the transducer function. Weibull 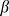 is found by multiplying these two factors together. We derive general analytical formulae for both factors, and then derive, from these formulae, specific expressions for 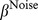 for a variety of noise distributions, and specific expressions for 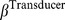 for several commonly used transducer functions.

Our work greatly extends a result previously published by Pelli [19]. He showed that, for 2AFC detection or discrimination,
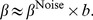
(7)where 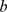 is the slope of 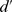 [20] against 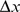 on log-log axes. Pelli derived this relationship using the concrete example of *contrast detection*, but it is a purely mathematical relationship (outlined in his “Analysis” section, Ref. [19], p. 121), which makes no assumptions about the underlying model, and could equally well be applied to *discrimination* along any unspecified stimulus dimension by replacing the contrast term, 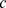, with 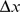 in his Equations (14) to (21).

Pelli's analysis ran as follows. Given the *definition* of 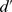 for 2AFC,
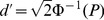
(8)(where 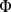 is the cumulative Gaussian), and the *observation* or *assumption* 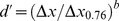 (where 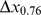 is the value of 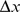 corresponding to a proportion correct of 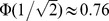, giving 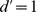, and 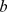 is the log-log slope of 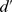 against 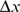), we have
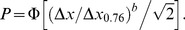
(9)Note that Equation (9) has the same form as Equation (6) if the pedestal, 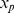, is zero, the transducer is a power function, 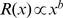, and the internal noise CDF is the cumulative Gaussian (as is usually assumed). If we let 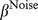 represent the 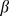 of the Weibull function, 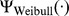, that fits best to the cumulative Gaussian, 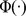, then, substituting this Weibull function for 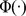 in Equation (9) yields a Weibull function with 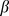 given by 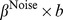, which is Relation (7).

In our terms, the “
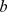
” part of Relation (7) is 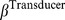, the factor determined by the transducer; we will show that, in the case of a power-function transducer and zero pedestal, our general expression for 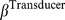 reduces to 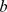. We obtain Weibull 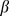 by multiplying 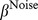 and 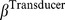 together, resulting in an estimated 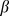 given by 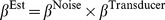, which is equal to 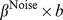 in the scenario just described. In this paper, we derive general analytical expressions for 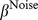 and 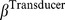 so that we can easily estimate Weibull 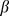 for any combination of noise distribution and transducer function, not just the specific case considered by Pelli.

In many situations, the observer can be modelled using a linear filter. This is equivalent to using a linear transducer, 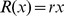, where 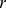 is a constant. For this transducer, Equation (6) gives
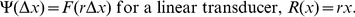
(10)Thus, the linear observer's psychometric function (plotted on a linear abscissa, 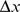) will have the same basic shape as the internal noise CDF, 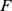, just differing by a horizontal scaling factor, 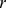. So, if the observer behaves in a linear fashion, the psychometric function plotted on linear axes gives us a direct plot of the shape of the internal noise CDF. In this situation, since 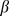 controls the Weibull function's shape on linear axes, the 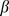 that fits best to the psychometric function will be the 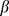 that fits best to the noise CDF (the sensitivity parameter, 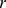, will determine the best-fitting 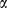, since 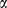 controls the Weibull function's horizontal scaling on linear axes).

The internal noise is usually assumed to be Gaussian, but Neri [18] has recently disputed this assumption. Using reverse correlation methods, he attempted to measure both the “deterministic transformation” (in our terms, the transducer function for contrast), and the shape of the internal noise distribution. He concluded that, for temporal 2AFC detection of a bright bar in noise, the contrast transducer was linear, and the internal noise had a Laplace distribution (whose CDF and PDF are given in Figures 4A and 4E, respectively). This is a radical departure from the Gaussian assumption that has usually been made since the invention of signal detection theory in the 1950s [14]. The Laplace distribution has higher kurtosis (i.e., has a sharper peak and heavier tails) than the Gaussian (compare Figure 4E with 4F). As we shall see later on, for positive 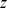, the Laplace distribution has a CDF that takes the form of a Weibull function with 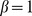. Since the psychometric function has the same shape as the internal noise CDF for a linear observer, Neri's proposal that the transducer is linear and the internal noise has a stimulus-independent Laplace distribution predicts that the observer's psychometric function should, like the Laplace CDF, be a Weibull function with 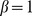. As noted earlier (and shown in Table 1 and Figure 1), this does not generally seem to be the case – with noise-free stimuli, 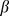 is around 3 for contrast detection and, even for suprathreshold contrast discrimination, where 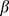 is substantially lower, it is still usually found to be greater than 1; later, we shall show that, assuming additive noise, these 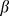 values are more consistent with a distribution that has *lower* kurtosis than a Gaussian.

Although the whole of this paper is couched in terms of the transducer model, it is not necessary to accept the transducer model to find the results useful; we just have to assume that the psychometric function has a form *consistent* with a particular combination of internal noise distribution and transducer function. For example, the intrinsic uncertainty model produces psychometric functions that are *consistent* with additive noise following an expansive power-function transducer with exponent that increases with channel uncertainty [21], but the model itself has no explicit transducer. Alternatively, suppose the observer carries out the discrimination task by making noisy estimates of each stimulus value and comparing them. Due to the noise, repeated presentations of the same stimulus value, 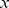, will give a distribution of estimated values, 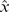, around the mean estimate. If we can find a function, 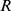, such that the shape and width of the distribution of 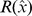 is independent of 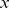, then the observer is equivalent to a transducer model with additive noise. In this class of model, the stimulus value, 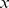, is transduced to give 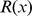, and then stimulus-independent noise is added to the signal. But we do not have to assume that this is literally how the observer works – the noisy estimates of the stimulus values could have arisen from all sorts of mechanisms, not just a transducer followed by additive noise.

In keeping with our terminology of 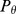 for the threshold performance level, we introduce the terms 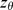 and 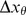 to represent the values of 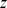 and 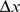 at threshold, i.e. the values of 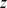 and 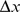 when the proportion correct is 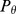, which we define as 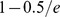.

## Theorem 1: A General Expression for the Psychometric Function in Terms of the Stimulus Values and the Threshold

### Introduction

Equation (6) gives a general equation for the psychometric function in terms of the transducer function, 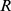, and the noise CDF, 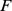. The sensitivity of the system (which determines the discrimination threshold, 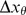) can be adjusted either by changing the gain of the transducer function (i.e., stretching or compressing 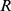 along its vertical axis), or by adjusting the spread of the noise CDF (i.e., stretching or compressing 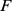 along its horizontal axis), or both. Since the units in which we express the internal signal are arbitrary, researchers will usually either (1) fix the spread of the noise CDF at some convenient standard value (say, unit variance), and vary the transducer gain to achieve the desired threshold, or (2) fix the gain of the transducer at some convenient standard value (say, unit gain), and vary the spread of the noise CDF to achieve the desired threshold. However, for our purposes, it is more convenient to reformulate Equation (6) so that *both* the spread of the noise CDF *and* the gain of the transducer are set to convenient values, and the threshold is specified directly. This allows us to consider general forms of the transducer and noise, without having to worry about specifying the gain of the transducer or spread of the noise correctly – the reformulated equation will take care of the spread of the psychometric function automatically. Theorem 1 derives an expression for the psychometric function that meets these requirements.

### Statement of Theorem 1

Theorem 1 has three parts:

1. The expression for the psychometric function, 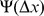, in Equation (6) can be rewritten as
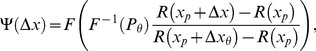
(11)where 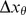 is the stimulus difference corresponding to a performance level of 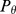.
2. If we change the gain of the transducer by replacing the function 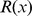 with 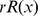, this will have no effect on the psychometric function, 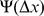, in Equation (11).
3. Similarly, if we change the spread of the noise CDF by replacing the function 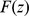 with 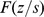, this will have no effect on 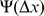 in Equation (11).

### Proof of Theorem 1

First, let us substitute the threshold values of 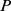 and 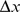 into Equation (6):
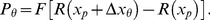
(12)
Equation (12) can be rearranged to give
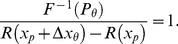
(13)Since the left hand side of Equation (13) is equal to 1, we can multiply anything by this expression, and leave it unchanged. Multiplying the argument of 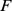 in (6) by this expression, we obtain Equation (11), which proves Part 1 of the theorem. If we replace the transducer, 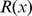, in Equation (11) with one that has a different gain, 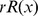, the 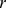
's will obviously cancel out, leaving the psychometric function, 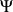, unchanged, which proves Part 2 of the theorem. To prove Part 3 of the theorem, consider what happens if we replace the function, 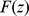, in Equation (11) with one that has a different spread, 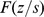. Then the inverse function is given by 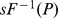, and the 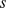
's cancel out:
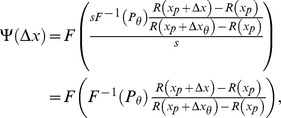
which is identical to Equation (11).□

### Discussion of Theorem 1

Equation (11) gives us an expression for the psychometric function (parameterized by the threshold, 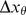) in which we can use any convenient standard form of the transducer function or noise distribution, without having to worry about setting the right gain or spread.

Although, for most of this paper, we define the threshold as the stimulus difference that gives rise to a performance level, 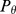, defined as 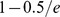, Theorem 1 actually holds for any value that 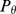 could have taken.

Note that, in the special case of a zero pedestal (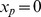) and a transducer that gives zero output for zero input (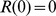), Equation (11) reduces to
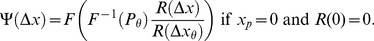
(14)


## Theorem 2. An Expression That Estimates the Best-Fitting Weibull *β* for Unspecified Noise and Transducer

### Statement of Theorem 2

Theorem 2 has two parts:

1. If the parameters of the Weibull function, 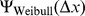, of Equation (1) can be set to provide a good fit to Equation (6), then the best-fitting beta will be well approximated by
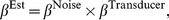
(15)where 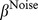 and 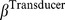 are given by the following expressions:
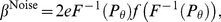
(16)

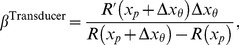
(17)and 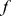 and 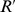 are the derivatives of, respectively, 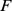 and 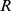 with respect to their inputs.

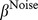
 is an estimate of the 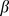 of the Weibull function that fits best to the noise CDF, 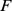, in Equation (6).

### Proof of Theorem 2

By assumption, the Weibull function provides a close fit to 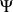 of Equation (6), so the gradient of 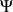 at threshold will closely match the gradient of the best-fitting Weibull function at threshold. Therefore, since 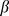 is proportional to the gradient of the Weibull function at threshold with an abscissa of 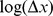, we can derive a close approximation to 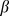 from the gradient of 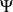 at threshold on this abscissa. To create a log abscissa, let 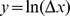, so that
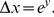
(18)If we substitute 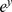 for 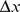 in Equation (1), we find that the gradient of the Weibull function on the log abscissa, 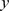, is given by
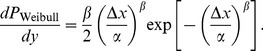
(19)For the Weibull function at threshold performance (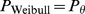), it follows that 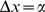. Substituting 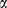 for 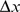 in (19) gives
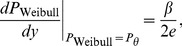
(20)and so
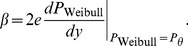
(21)To evaluate Equation (21), we use the chain rule to expand the derivative:
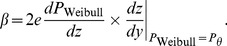
(22)As noted above, the assumed good fit of the Weibull function, 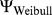, of Equation (1) to 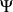 of Equation (6) means that the output, 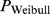, of the Weibull function is close to the output of 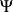, which is the proportion correct, 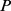. Substituting 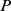 for 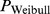 in Equation (22) therefore gives us a good estimate of 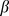, which we call 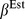:
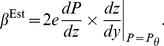
(23)From Equation (6), we see that 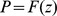, so 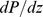 is given by 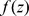, the noise PDF (which is the derivative of 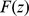 with respect to 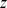). At threshold, 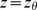, and so,
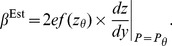
(24)We will see that the first part of Equation (24), 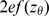, is proportional to 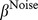, the 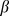-estimate of the Weibull function that fits best to the noise CDF, and the second part, 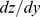 at threshold, is proportional to 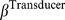 defined above. Most of the work involves deriving an expression for 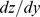 at threshold.

Using Equation (18) to substitute for 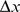 in Equation (3), we get
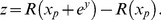
(25)Let us define 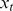 as the target stimulus value:
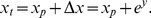
(26)Using Equation (26) to substitute for 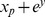 in Equation (25), we have
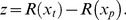
(27)Then,
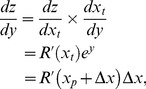
(28)where 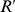 is the derivative of 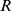 with respect to its input. At threshold, we can substitute 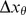 for 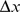 in Equation (28), giving
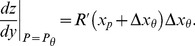
(29)Using Equation (29) to substitute for 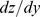 in Equation (24), we obtain
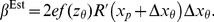
(30)From Equation (6), we have 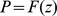, so, considering the values of 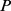 and 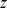 at threshold,
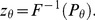
(31)Using Equation (31) to substitute for 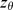 in Equation (30), we have
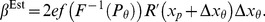
(32)To evaluate this expression as written in Equation (32), we need to know the gain of the transducer and the spread of the noise CDF, or at least their ratio. However, if we know the shape of the transducer (apart from the gain), and we know the shape of the noise CDF (apart from the spread), we can work out the ratio of gain to spread from 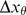. But it is much more convenient to reformulate Equation (32) so that this is taken care of, and we can arbitrarily set the spread of the noise CDF and the gain of the transducer to any convenient values. We can use the same trick that we used in Theorem 1: We multiply the expression in Equation (32) by the left hand side of Equation (13), which equals 1. After doing this, and rearranging the terms, we obtain
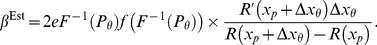
(33)
Equation (33) can be written in the form given by Equations (15) to (17), which proves the Part 1 of the theorem.

We now prove Part 2, that 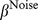 is the estimated *β* of the Weibull function that fits best to the noise CDF, 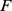. First, note that all linear transducers have the form 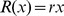. This gives 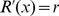, and so, from Equation (17), 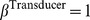, regardless of the value of 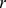, 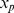 or 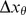. Therefore, from (15), 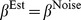 for a linear transducer. Now, consider the linear transducer 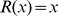. For this transducer, Equation (6) gives 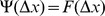. The estimate of *β* when the Weibull function, 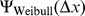, is fitted to 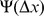 is given by 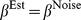, as it will be for any linear transducer. Since, in this case, 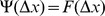, the Weibull function has also been fitted to the noise CDF, and the estimated 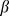 of this fitted function is given by 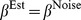.□

### Discussion of Theorem 2

To get an intuition into how Weibull 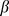 is partitioned into the two terms, 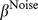 and 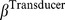, let us refer back to Equation (21). This equation shows that 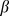 is proportional to 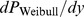 at threshold. We used the chain rule to express 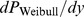 as 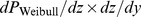, which is approximately equal to 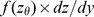. 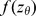 depends only on the noise distribution, and is proportional to 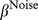; 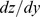 at threshold generally depends on the transducer, the pedestal and the threshold, and is proportional to 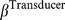; their product is proportional to Weibull 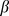. This is essentially where Equations (15) to (17) come from. The equations were tidied up by specifying the constants of proportionality, and defining 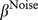 and 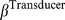 in such a way that they are independent of any horizontal scaling of the noise distribution, or any vertical scaling of the transducer function. Thus, the 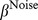 term will be the same for, for example, all Gaussian distributions, whatever the spread, and the 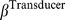 term will be the same for, for example, all power functions with a particular exponent, whatever the gain.

Equation (17) expresses 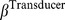 as a function of the threshold stimulus difference, 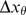. Alternatively, for nonzero pedestals, we can reformulate Equation (17) as a function of the Weber fraction, 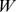, defined as the ratio 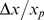 at threshold:
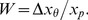
(34)From Equation (34), we obtain 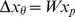, and, using this expression to substitute for 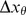 in Equation (17), we can rewrite the expression for 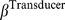 in terms of 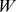:
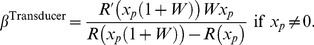
(35)


The Weber fraction can only be defined if 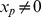. If 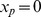 and 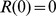, Equation (17) reduces to
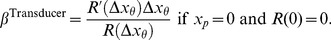
(36)When the stimulus dimension of interest is contrast, a discrimination experiment with a zero pedestal is called a contrast detection experiment.

**Figure 5:**
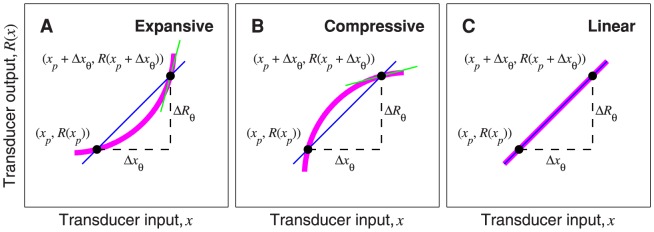
Geometrical interpretation of the expression for 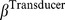. In each panel, the thick, magenta curve represents the transducer function. The horizontal axes represent the transducer input, and the vertical axes represent the transducer output. 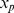 is the pedestal level, and 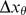 is the discrimination threshold. The gradient of the blue line, 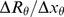, is equal to 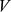, defined in Equation (39). The green line is the tangent to the transducer at point 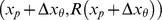; its gradient is equal to 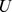, defined in Equation (38). The ratio 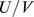 is equal to 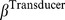. For an expansive transducer (panel A), 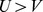, so 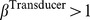. For a compressive transducer (panel B), 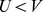, so 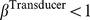. For a linear transducer (panel C), 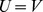, so 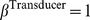.

One important property of 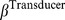 is that it is always greater than 1 for a fully expansive transducer function (i.e., one for which the slope always increases away from zero with increasing input), and is always less than 1 for a fully compressive transducer function (i.e., one for which the slope always decreases towards zero with increasing input). Here we provide a geometrical argument (illustrated in Figure 5) to explain why this is the case.

First, note that we can rewrite Equation (17) as
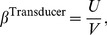
(37)where
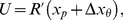
(38)and
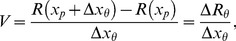
(39)with
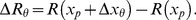
(40)


These quantities are illustrated for an expansive transducer in Figure 5A, where the thick, magenta curve represents the transducer. The filled circles mark points 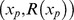 and 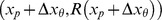. The gradient of the blue line connecting these two points is 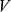, defined in Equation (39). The short, green, line segment is the tangent to the transducer at 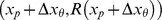; its gradient is 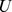, defined in Equation (38). It is clear from the diagram that, for an expansive transducer, like the one illustrated, the gradient of the transducer at 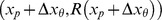 must always be steeper than the blue line, because, as we travel along the transducer function from 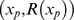 to 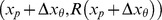, the transducer approaches the second point from below the blue line. Therefore, 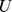 must always be greater than 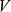, so, from Equation (37), 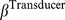 must always be greater than 1.

Figure 5B illustrates the situation for a compressive transducer. Here, as we travel along the transducer function from 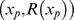 to 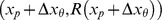, the transducer approaches the second point from above the blue line, and so the gradient of the transducer at the second point must be lower than the gradient of the blue line. Thus, 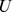 must always be less than 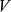, so, from Equation (37), 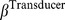 must always be less than 1.

Finally, Figure 5C illustrates the situation for a linear transducer, i.e. one that is neither expansive nor compressive. Here, the gradient of the transducer is equal to the gradient of the blue line, so 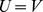, and therefore 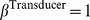. This provides a geometrical insight into the previously proved fact that 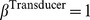 for a linear transducer.

In conclusion, Weibull 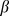 can be partitioned into two factors: 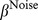 (Equation (16)), which estimates the 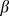 of the Weibull function that fits best to the noise CDF, 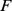; and 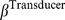 (Equation (17), (35) or (36)), which is determined partly (or, as we shall see, sometimes completely) by the shape of the transducer function, 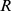. 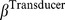 is greater than 1 for an expansive transducer, less than 1 for a compressive transducer, and equal to 1 for a linear transducer. 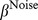 is independent of the spread (i.e. horizontal scaling) of the CDF (analogously, Weibull *β* is independent of the spread of the Weibull function on linear axes); 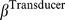 is independent of the gain (i.e. vertical scaling) of the transducer. Multiplying 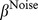 and 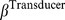 together gives us 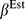, the estimate of Weibull 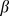. The expressions for 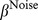 and 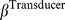 derived above are completely general. In later sections, we derive values for 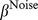 given specific noise distributions, and expressions for 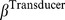 given specific transducers.

There are two possible sources of error in the Weibull 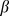 estimate, 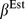. Firstly, the derivation of the expression for 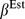 relies on the use of 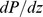 as an approximation of 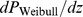 at threshold in the step from Equation (22) to (23), where 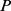 is the output of the psychometric function, 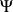, and 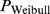 is the output of the best-fitting Weibull function. The accuracy of 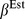 relies on these two slopes being close at the threshold performance level. A second potential source of inaccuracy is that, even if these two slopes are very close at the threshold level, the overall psychometric function, 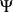, might still not be well fit by a Weibull function, in which case the best-fitting Weibull 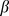 could deviate substantially from 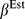. However, as we will show, in the range of conditions usually encountered, the Weibull function does provide a good fit to the psychometric function, so 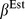 is accurate. In cases where 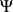 is a Weibull function, the best-fitting Weibull function will fit exactly, and 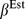 gives the exact value of the best-fitting Weibull 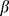.

## Deriving 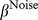 for Specific Noise Distributions

As proved in Theorem 2, 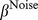 is an estimate of the 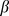 of the Weibull function that fits best to the noise CDF. In this section, we evaluate the analytical expression for 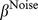 (Equation (16)) for several different noise distributions. We also compare each value with the 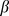 value obtained by fitting the Weibull function to the noise CDF numerically. There is of course no single correct answer to the question of what is the best-fitting 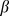 – it depends on both the fitting criterion and the points on the psychometric function that are sampled. When Pelli [19] fitted the Weibull function to the Gaussian CDF, he minimized the maximum error over all positive inputs. We instead performed a maximum-likelihood fit over all inputs from 0 to twice the threshold (actually, we approximated this by sampling the psychometric function in discrete steps of one thousandth of the threshold). Our rationale for this approach was that fitting the psychometric function is usually done by maximum likelihood, and the threshold usually falls around the middle of the set of stimulus values.

### Evaluating 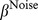 for a generalized Gaussian noise CDF

Most psychophysical models use Gaussian noise. This is partly because the Gaussian is often easy to handle analytically, but also because, according to the Central Limit Theorem, the sum of independent sources of noise tends towards a Gaussian-distributed random variable, whatever the distribution of the individual noise sources. However, as noted earlier, Neri [18] has recently argued that internal sensory noise is closer to a Laplace distribution. Both the Gaussian and the Laplace are parameterizations of the generalized Gaussian, which we consider in this section.

The generalized Gaussian CDF is given by the following expression, with horizontal scaling (i.e. spread) determined by 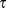, and shape determined by 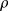:
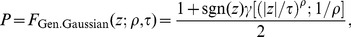
(41)where 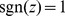 for 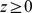 and 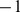 for 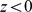, and 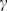 is the lower incomplete gamma function, defined as
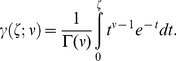
(42)

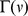
 in Equation (42) is the gamma function, which is a continuous generalization of the factorial, given by
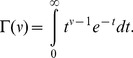
(43)Note, the lower incomplete gamma function is often defined without the normalization term, 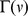, but it is more convenient for us to define it as in Equation (42), because otherwise we would just have to divide by 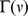 anyway, complicating the expression for the generalized Gaussian in Equation (41); in addition, the MATLAB function gammainc evaluates the function as defined in Equation (42).

The variance of the generalized Gaussian distribution is given by
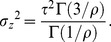
(44)We use the subscript, 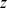, in Equation (44) to indicate that this is the variance of the noise on the difference of mean signals, 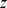, as opposed to the variance of the noise on the transducer outputs, which we could call 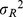. As long as the noise on the two transducer outputs within a trial is uncorrelated and has zero mean, then we have 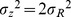, and so 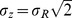, whatever form the noise CDF takes.

The PDF of the generalized Gaussian distribution is given by the derivative of the CDF:
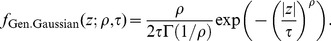
(45)As noted above, the shape of the distribution is determined by the parameter, 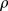. When 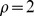, Equation (45) describes the Gaussian PDF:
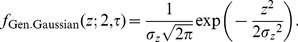
(46)When 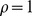, Equation (45) describes the Laplace PDF:
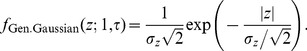
(47)For positive 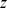, the inverse of the generalized Gaussian CDF is given by
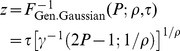
(48)(we don't need to worry about negative 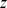, because, for any monotonically increasing transducer, and positive 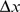, 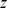 as defined in Equation (3) is always positive). The inverse of the lower incomplete gamma function, 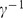, in Equation (48) can be evaluated using the MATLAB function gammaincinv. At threshold, 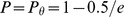. Substituting these values into Equation (48), we get
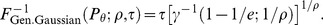
(49)We can use the expression for 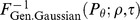 in Equation (49) to substitute for 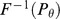 in Equation (16), and we can use the expression for 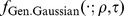 in Equation (45) to substitute for 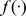 in Equation (16). The different instances of 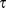 cancel out, giving us an expression for 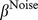 for the generalized Gaussian noise distribution that is a function of 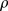:
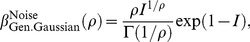
(50)where
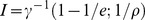
(51)The subscript, “Gen.Gaussian”, on 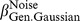 in Equation (50) indicates the general form of the noise CDF.

**Figure 6:**
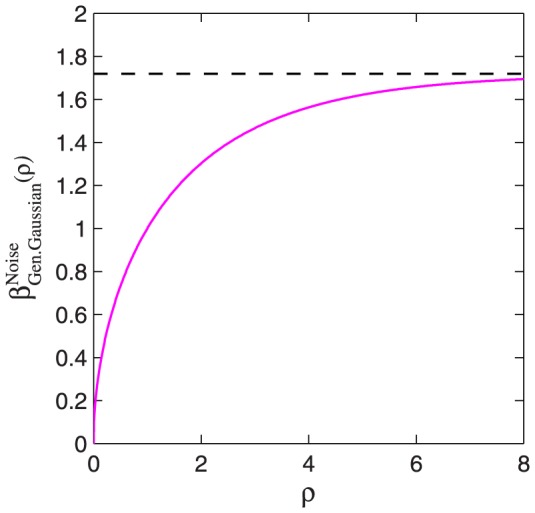
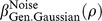
 plotted as a function of 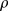. This curve plots the predicted 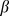 when the Weibull function is fitted to the CDF of generalized Gaussian distributions with a range of different 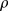 values. The graph asymptotes to a value of 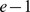 (see Appendix S1), indicated by the horizontal dashed line. The shape of the generalized Gaussian distribution is determined by 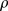. 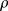-values of 1 and 2 are special cases: 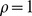 gives a Laplace distribution, and 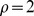 gives a Gaussian distribution.

Figure 6 plots 
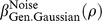
 as a function of 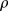. As proved in Appendix S1, 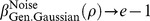 as 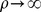. Values of 
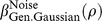
 for 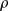 = 1, 2, and 4 are given by
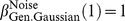
(52)

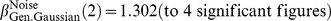
(53)

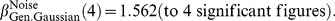
(54)The value of 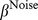 for the Laplace distribution (Equation (52)) is exactly 1. This is because the positive half of its CDF is a Weibull function with 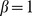. This can be seen from the fact that 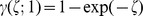, and so Equation (41) gives, for positive 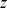,
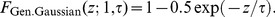
(55)The Weibull function with 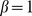 therefore gives an exact fit to the Laplacian noise CDF, and the estimated Weibull 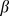, given by 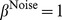, is exactly correct.

**Figure 7:**
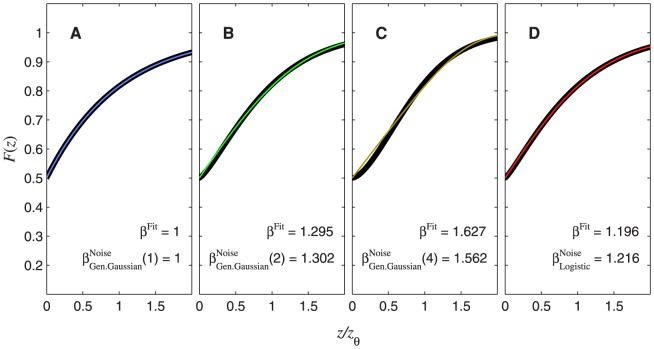
Noise CDFs from Figure 4 plotted against the best-fitting Weibull functions. The thin, coloured curves shown in (A) to (D) are the CDFs from Figures 4A to 4D, respectively. The thick, black curves are the Weibull functions that give the best (maximum-likelihood) fit across the range of inputs shown on the horizontal axis. This fit was carried out by maximizing the expression 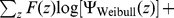 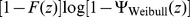, where 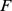 is the noise CDF, and 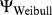 is the Weibull function whose parameters were being fitted. The Weibull function provides a perfect fit to the Laplace CDF (A), an excellent fit to the Gaussian (B), and logistic (D) CDFs, and an acceptable fit to the generalized Gaussian with 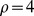 (C); this partly justifies our use of 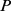 as an estimate of 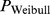 in Equation (23). The 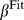 values are the 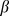 parameters of the fitted Weibull functions. The 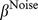 values are our analytical estimates of 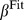, given by Equations (52) to (54) for panels (A) to (C), respectively, and Equation (60) for panel (D). In each case, 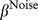 provides a close match to 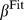. The parameter in brackets in each 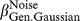 term is the shape parameter, 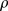 (see Equation (50)). As noted in the text, the CDFs all have a point of inflection at zero. With the exception of panel A, the best-fitting Weibull functions have a point of inflection slightly above zero (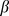 would have to be 1 or less for the steepest point to occur at zero). Nevertheless, the Weibull functions still provide good fits.

The coloured curves in Figures 7A, 7B, and 7C show the generalized Gaussian noise CDFs for 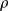 = 1, 2, and 4, respectively, and the thick, black curves show the best-fitting Weibull functions (maximum-likelihood fit over inputs from 0 to twice the threshold). Also shown in each panel is the appropriate value of 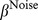 from equations (52) to (54), and the best-fitting Weibull 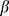, which we call 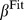. As explained above, the match between 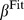 and 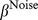 is exact for the Laplace (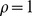, Figure 7A), but the match is also very good for the other distributions. For the Gaussian (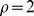, Figure 7B), 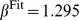, very close to our analytically derived value of 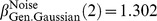. As discussed earlier, Pelli [19] fitted the Weibull to a Gaussian CDF using a different fitting method: He minimized the maximum error over all positive inputs. The 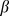 value he obtained from this fit was 1.247. As noted earlier, there is no single “correct” answer, but our maximum-likelihood fitting paradigm is probably more representative of the process of fitting a function to psychophysical data, and our obtained 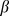 of 1.295 is very close to the analytically obtained value. The match between 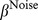 and 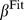 for 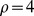 (Figure 7C) is also close, the deviation being far smaller than the margin of error usually encountered when measuring Weibull 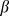 [22]–[27].

### Evaluating 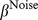 for the logistic noise CDF

Sometimes, the logistic function is used instead of the Gaussian, for computational convenience (e.g. Ref. [28]). The logistic function is very similar in shape to the Gaussian. Its CDF is given by
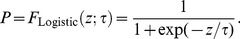
(56)As noted by Strasburger [29], this function is identical to the hyperbolic tangent function, given by 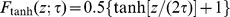. Its variance is 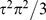. The PDF of the logistic distribution is given by the derivative of the CDF:
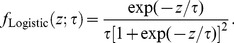
(57)The inverse of the logistic CDF is given by
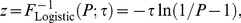
(58)At threshold, 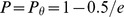. Substituting these values into Equation (58), we get
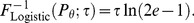
(59)We can use the expression for 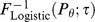 in Equation (59) to substitute for 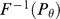 in Equation (16), and we can use the expression for 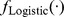 in Equation (57) to substitute for 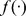 in Equation (16). This gives
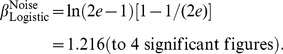
(60)As before, the subscript on 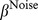 indicates the form of noise CDF. The accuracy of this approximation is confirmed in Figure 7D. The 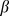 parameter of the fitted Weibull function (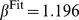) is very close to the estimated value from Equation (60).

## Theorem 3. Tendency towards Linear Behaviour with Non-Zero Pedestals

### Introduction

As shown earlier, for a linear transducer, 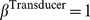, and so 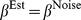, which takes a value of around 1.3 for Gaussian internal noise. So, if a transducer model has additive Gaussian noise and generates psychometric functions with a Weibull 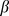 of about 1.3, that might seem to suggest that it contains a linear transducer. However, a transducer model with additive Gaussian noise can in fact generate psychometric functions with 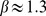 for suprathreshold contrast discrimination even when the transducer departs wildly from a linear function [4]. Theorem 3 explains how this occurs.

### Statement of Theorem 3

If the gradient of the transducer is not 0 or 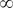 at the pedestal level, then, as 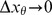, 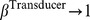.

### Proof of Theorem 3

As noted earlier, 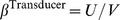, where 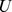 and 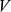 are given in Equations (38) and (39), respectively. The limit of 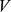 as 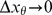 is the derivative of 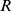 at 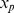, i.e.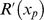, by definition of the derivative, and the limit of 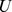 as 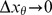 is obviously 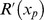, so we have
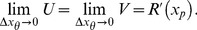
(61)Then, provided that 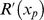 is not 0 or 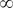, we have
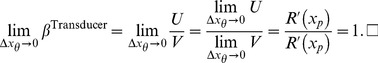
(62)If 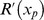 is 0 or 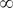, then 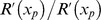 is an indeterminate form, 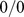 or 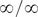, and cannot be evaluated. In this case, we cannot evaluate the limit of 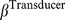 by dividing the limit of 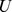 by the limit of 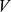. The limit must instead be evaluated in some other way that will depend on the form of the transducer, and the limit in this case will not necessarily be 1.

### Discussion of Theorem 3

Theorem 3 shows that, *whatever* the transducer function, as long as its gradient is not 0 or 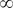 at the pedestal level, Weibull 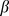 will approach that for a linear transducer as sensitivity improves. Virtually all proposed transducers do have a finite, nonzero gradient for nonzero inputs; therefore, if the internal noise is approximately Gaussian, we would expect Weibull 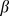 to be close to 1.3 for suprathreshold contrast discrimination. Detection and discrimination data are often fitted with a power-function transducer or a Legge-Foley transducer (both considered below), and, with these transducers, the gradient is 0 or 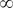 at an input level of zero. Thus, for these transducers, when the pedestal level is zero, Equation (62) does not apply, and 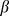 does not necessarily approach that for a linear transducer as sensitivity improves. This explains why, for contrast detection experiments (i.e. when the pedestal is zero), Weibull 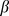 has been found to deviate greatly from the value of 1.3 expected from a linear transducer with Gaussian noise.

Consider what happens in general when the pedestal approaches zero. If we assume that 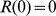, then, as 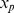 drops below 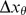, both 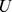 (Equation (38)) and 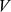 (Equation (39)) become dominated by the 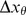 term, and 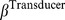 approaches the value given in Equation (36), which is not, in general, equal to 1. Thus, we would expect Weibull 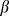 to deviate substantially from the linear case for low pedestals. Meese, Georgeson and Baker [4] showed that this is indeed the case for visual contrast discrimination, and we examine their work in more detail later, in the section on the Legge-Foley transducer.

## Deriving Psychometric Functions and Weibull *β* for Specific Nonlinear Transducers

As shown earlier, 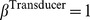 for any linear transducer. For a nonlinear transducer, 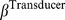 will deviate from 1, and this is how the transducer has its effect on 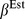, the estimated Weibull 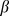. Starting with one of the general expressions for 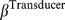 (Equation (17), (35) or (36), as appropriate), we can substitute a specific transducer function for the general function, 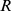, to give a specific expression that describes 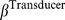 for that transducer. Similarly, starting with one of the general expressions for the psychometric function (Equation (11) or (14), as appropriate), we can substitute a specific transducer function for the general function, 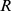, to give a specific expression for the psychometric function. In this section, we consider five commonly used scenarios: a power function with zero or nonzero pedestal, a log function, and a Legge-Foley function [30] with zero or nonzero pedestal.

Power-function transducers have been used to account for visual contrast discrimination data. As the pedestal increases from 0, the discrimination threshold first decreases, and then starts to increase with further increases in pedestal; this function, giving contrast discrimination threshold at each pedestal level, is known as a “dipper function”. The initial dip can be explained by an expansive power function (i.e., one with exponent greater than 1) at low contrasts [1], [6], while the increase in contrast discrimination threshold for larger pedestals can be explained by a compressive power function (i.e., one with exponent less than 1) at high contrasts. The Legge-Foley transducer approximates an expansive power function at low contrasts and a compressive power function at high contrasts, and accounts for the whole dipper function [4], [30]. We also include the log transducer in our analysis, firstly because discrimination at high pedestal levels has often been found to adhere closely to Weber's law in many different perceptual dimensions and sensory modalities [31]–[35] (a prediction of the log transducer with additive noise), and, secondly, because we have discovered an interesting link between the power function and the log transducer, which is presented in Theorem 4.

### Power function and zero pedestal

The first case that we consider is the one examined by Pelli [19], i.e. 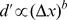. As noted earlier, this relationship between 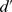 and 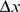 is consistent with a power function transducer (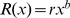) and zero pedestal (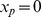). In this case, we can use Equation (36) to derive 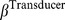, and it follows easily that
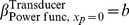
(63)(as with 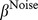, the subscript on 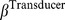 describes the specific case). Thus, using Equation (63) to substitute for 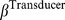 in Equation (15), we have
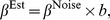
(64)which is the relationship derived by Pelli [19] (Relation (7) of this paper).

**Figure 8:**
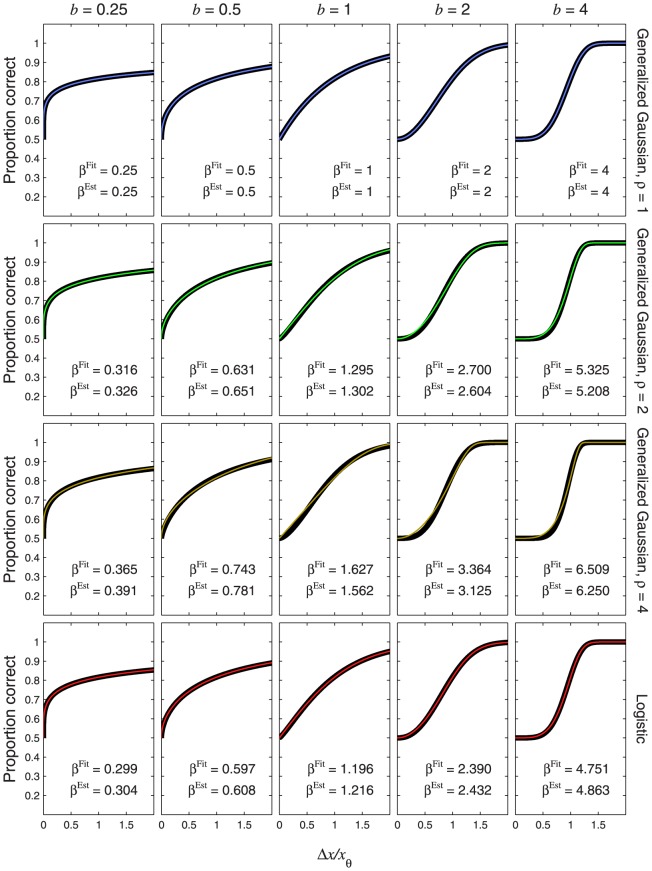
Psychometric functions resulting from power-function transducers and zero pedestal. The thin, coloured curves show the psychometric function of Equation (65), plotted as a function of 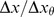. Different rows of panels show psychometric functions with different noise CDFs, 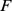, given by the Laplace distribution (top row of panels), the Gaussian (second row), the generalized Gaussian with 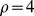 (third row) or logistic (bottom row). Different columns of panels show psychometric functions for different transducer exponents, 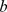, as indicated at the top of the figure. The thick, black curves show the best-fitting (maximum-likelihood) Weibull functions. The curves in the middle column (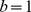, top to bottom) are identical to Figures 7A to 7D, respectively. This is because 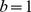 gives a linear transducer, and so the psychometric functions for 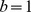 will have the same shape and same fitted 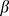 as the CDF (see Equation (10)). Each panel displays the 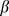 value of the best-fitting Weibull function (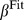) and the estimate, 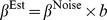, where 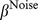 is given by Equation (52), (53), (54) or (60), as appropriate.

From Equation (14), it follows that, for a power function transducer and zero pedestal, the model's true psychometric function is given by
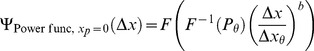
(65)with the subscript on 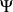 describing the specific case. Equation (65) gives us the option of expressing the stimulus difference in absolute units, 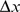, or in “threshold units”, 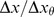 – the two options differ only in a linear horizontal scaling. The latter is useful when dealing with general cases where the threshold is not specified; the psychometric function is often expressed in this way [19], [29], [36]. The coloured curves in Figure 8 show the psychometric function of Equation (65). Different rows of panels show psychometric functions for different noise CDFs, 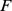, as indicated on the right of the figure. Different columns of panels show psychometric functions for different transducer exponents, 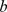. The thick, black curves show the best-fitting Weibull functions. These provide a good fit to the true psychometric functions, justifying the premise of Theorem 2, which is that the Weibull function provides a good fit.

Each panel of Figure 8 also compares 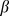 of the best-fitting Weibull function (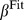) with the estimate, 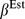, given by 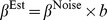. In every case, 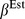 is very close to the fitted value, the discrepancy being far smaller than the margin of error usually encountered in psychophysical measurements of psychometric function slope [22]–[27]. For each transducer (i.e. each column of Figure 8), the difference in 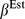 between the different noise CDFs (i.e. between the different rows of Figure 8) is caused entirely by the different values of 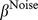. For example, the value of 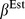 for the Gaussian will always exceed that for the Laplace by a factor 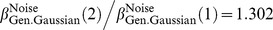.

### Power function and nonzero pedestal

**Figure 9:**
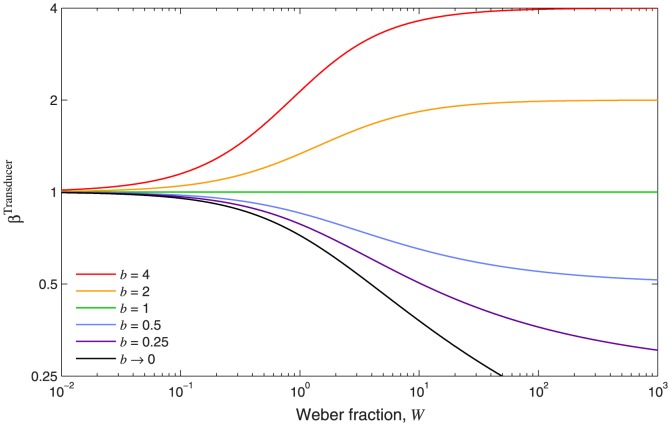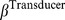 for the power-function transducer with nonzero pedestal, plotted as a function of Weber fraction. Each curve gives 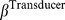 for a different transducer exponent, 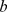. 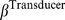 asymptotes towards 1 as 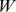 decreases, and towards 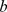 as 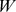 increases. For typical Weber fractions of less than 0.3 (see Table 1), 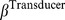 does not deviate much from 1. The bottom curve, in black, shows the limiting case, as 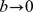. All the plotted functions except the one for 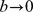 are given by Equation (67). In Theorem 4B, we prove that the limiting case as 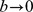 is identical to the curve corresponding to a logarithmic transducer; this curve is given by Equation (70).

We now consider the case of a power function and any pedestal value, a generalization of the previous case. First, starting with Equation (17), we trivially obtain 
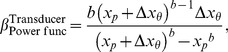
(66)which reduces to Equation (63) when 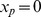. When 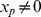, we can start with Equation (35), from which it follows straightforwardly that
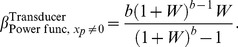
(67)
Figure 9 plots 
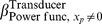
 as a function of the Weber fraction, 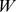 (defined in Equation (34)), for several values of the transducer exponent, 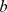. These curves all converge to a value of 1 towards the left. This is because, for a power function transducer with nonzero pedestal, the gradient of the transducer at the pedestal level is not 0 or 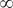, and so, as proved in Theorem 3, 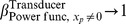 as 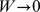.

**Figure 10:**
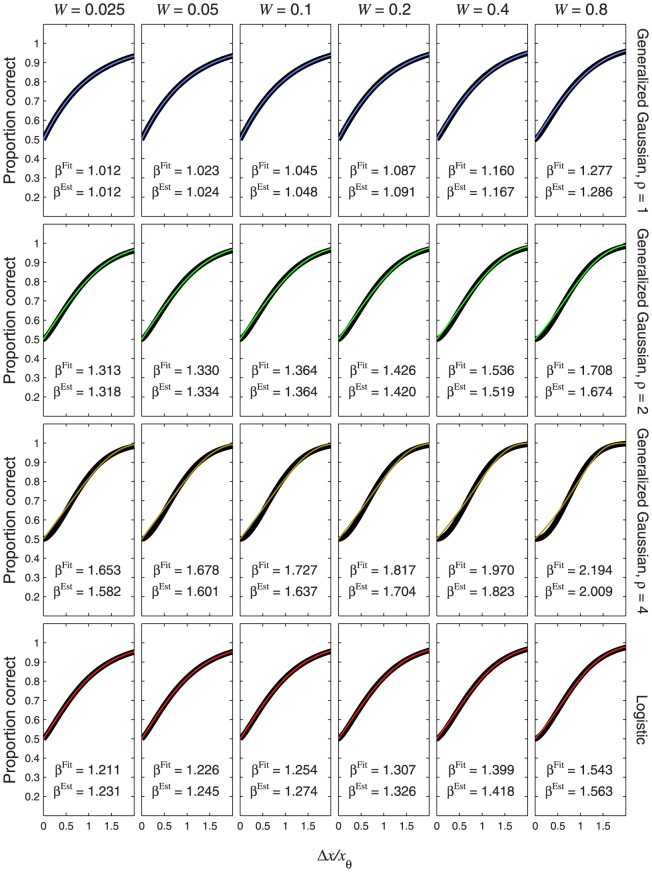
Psychometric functions resulting from transducer 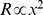 and nonzero pedestal. The thin, coloured curves show the psychometric function of Equation (69) with 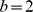. Different rows of panels show psychometric functions with different noise CDFs, as indicated on the right of the figure. Different columns of panels show psychometric functions for different Weber fractions, 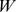. The thick, black curves show the best-fitting (maximum-likelihood) Weibull functions. Each panel displays the 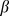 value of the best-fitting Weibull function (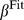) and the estimate, 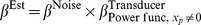, where 
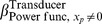
 is given by Equation (67), and 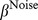 is given by Equation (52), (53), (54) or (60), as appropriate.

**Figure 11:**
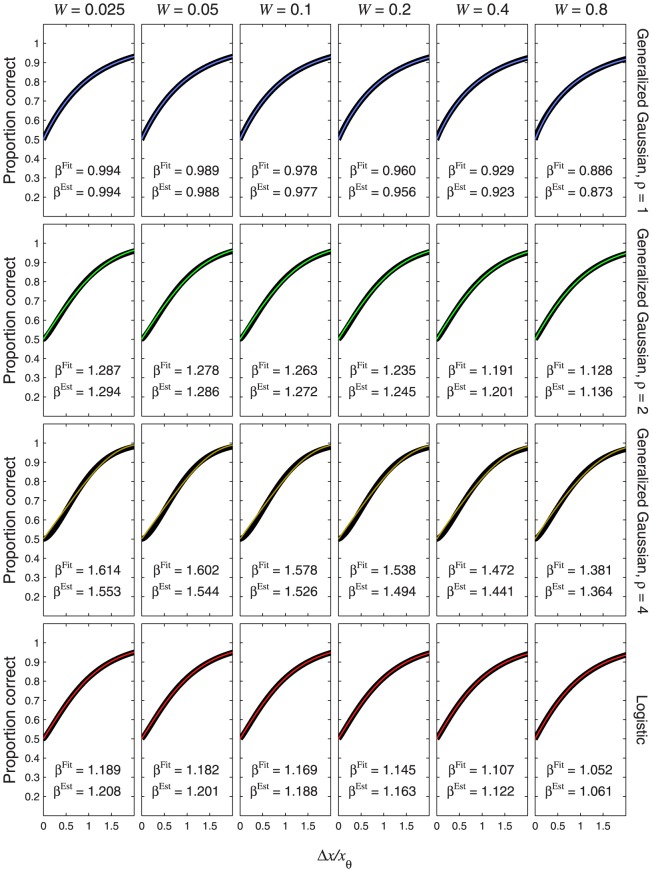
Psychometric functions resulting from transducer 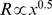 and nonzero pedestal. All details are the same as in Figure 10, except that the transducer exponent is 0.5.

From Equation (11), we can see that, for a power function transducer with unspecified pedestal, the model's true psychometric function is given by
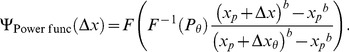
(68)If 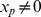, then we can divide through by 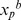, and rewrite Equation (68) in terms of the Weber fraction, 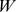:
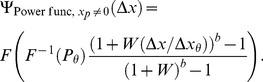
(69)
Equation (68) is a general formula for the psychometric function, given a power-function transducer and any pedestal value. Equations (65) and (69) are simpler expressions for this psychometric function in the cases of zero and nonzero pedestals, respectively. As with Equation (65), Equation (69) gives us the option of expressing the stimulus difference in absolute units, 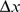, or threshold units, 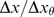. The coloured curves in Figure 10 show the psychometric function of Equation (69) for a range of Weber fractions and a transducer exponent of 2. Different rows of panels show psychometric functions for different noise CDFs, and different columns of panels show psychometric functions for different Weber fractions, 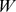. The thick, black curves show the best-fitting Weibull functions. Each panel also compares 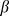 of the best-fitting Weibull function (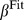) with the estimate, 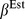, given by 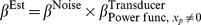. Figure 11 is the same as Figure 10 except that the transducer exponent is 0.5. In every case, the Weibull function fits well to the true psychometric function, and the agreement between 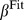 and 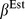 is very good.

It is interesting to compare the psychometric functions in Figures 10 and 11 with those for the same transducer when the pedestal is zero; these are given in the columns of Figure 8 headed “
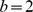
” and “
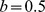
”, respectively. It is clear that, even with the rather large Weber fraction of 0.8, the existence of a nonzero pedestal brings Weibull 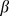 much closer to the linear case (the case of a linear transducer is shown in the column of Figure 8 headed “
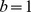
”).

### Logarithmic transducer

The log function is undefined for zero inputs, so we can only consider a log transducer for nonzero pedestals. If the transducer takes a logarithmic shape for all inputs greater than the pedestal value, then it is effectively logarithmic for the whole of the range of stimulus values being considered.

For a logarithmic transducer, 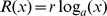, Equation (35) leads to
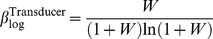
(70)for any base of logarithm, 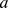. In Theorem 4B, below, we show that, for any Weber fraction, 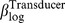 is the limiting value of 
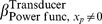
 as 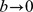. The bottom (black) curve in Figure 9 plots 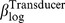 as a function of 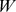.

**Figure 12:**
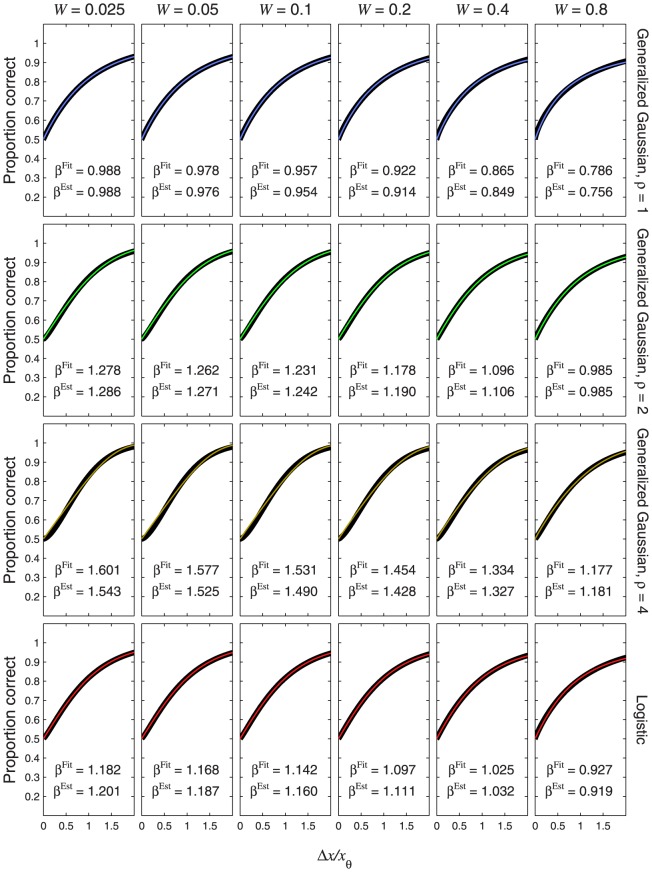
Psychometric functions resulting from a logarithmic transducer. The thin, coloured curves show the psychometric function of Equation (71). Different rows of panels show psychometric functions with different noise CDFs, as indicated on the right of the figure. Different columns of panels show psychometric functions for different Weber fractions, 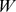. The thick, black curves show the best-fitting (maximum-likelihood) Weibull functions. Each panel displays the 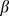 value of the best-fitting Weibull function (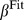) and the estimate, 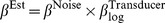, where 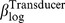 is given by Equation (70), and 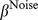 is given by Equation (52), (53), (54) or (60), as appropriate.

Starting with Equation (11), it is straightforward to show that, for a log transducer, the model's true psychometric function is given by
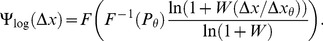
(71)The coloured curves in Figure 12 show the psychometric function of Equation (71) for a range of Weber fractions. Different rows of panels show psychometric functions for different noise CDFs, and different columns of panels show psychometric functions for different Weber fractions. The thick, black curves show the best-fitting Weibull functions. Each panel also compares 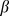 of the best-fitting Weibull function (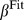) with the estimate, 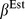, given by 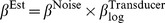. The Weibull function gives an excellent fit to the true psychometric function in every case, and the agreement between 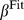 and 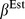 is very good. As the Weber fraction decreases, Weibull 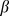 approaches that for the linear case.

### Legge-Foley transducer

If the noise is additive, then no single power-function transducer can fit contrast discrimination data across the whole contrast range, because we need an expansive function to explain facilitation at low contrasts, and a compressive function to explain the rise in threshold with pedestal at high contrasts. Legge and Foley [30] used a sigmoid transducer that was expansive at low contrasts and compressive at high contrasts, as required:
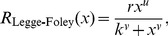
(72)where 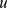, 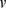, 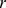 and 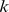 are constants, all greater than zero. The transducer in Equation (72) seems to have been first used in psychophysics by Stromeyer and Klein [37], and it is sometimes referred to as the Stromeyer-Foley function [36], [38], but we use the term “Legge-Foley transducer”, as Legge and Foley's use of this transducer is probably better known. For low inputs, 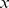, the Legge-Foley transducer approximates a power function with exponent 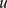; for large inputs, it approximates a power function with exponent 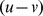. Legge and Foley had 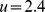 and 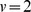, so the transducer was an accelerating power function (with exponent 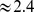) for low inputs, and a compressive power function (with exponent 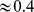) for high inputs. The point of inflection (at which the transducer changes from expansive to compressive) occurs at an 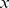 value close to 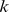 for typical values of the fitted parameters (see Appendix S2 for a derivation of the formula for calculating the position of the point of inflection).

Assuming a zero pedestal, we can substitute 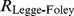 for 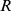 in Equation (36), giving
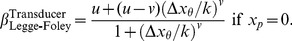
(73)If the threshold, 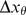, is much less than 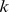, the right hand side of Equation (73) approaches 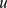, as we would expect: The Legge-Foley transducer in this case approximates a power-function transducer with exponent 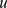, and, for the latter transducer with zero pedestal, 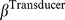 is simply equal to the exponent, as in Equation (63).

**Figure 13:**
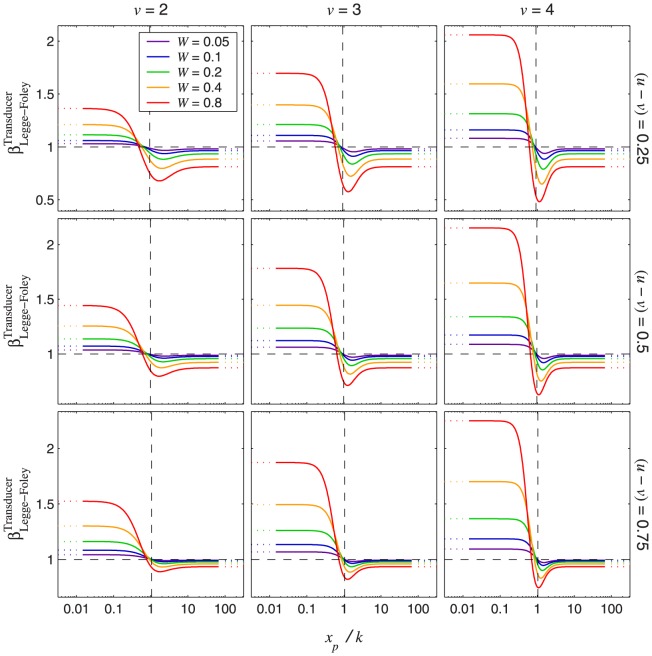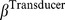 for the Legge-Foley transducer with nonzero pedestal. The curves were generated using Equation (74). Each column of panels has a particular value for 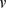, and each row of panels has a particular value for the difference 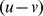. Within the panels, the Weber fraction, 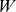, is indicated by the colour of the curve (the legend in the top-left panel applies to all panels). The curves approach horizontal asymptotes on the right (indicated by dotted lines), with vertical position given by Equation (67) with 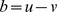. This is because, as mentioned earlier, as the input signal increases, the Legge-Foley transducer approaches a power function with exponent 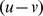. This asymptote can also be derived from Equation (74) by setting 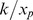 to 0, which gives the limit as 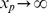. On the left, the curves come close to approaching an asymptote with vertical position given by Equation (67) with 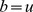 because, at low contrasts, the Legge-Foley transducer approximates a power function with exponent 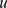. These near-asymptotes are indicated by dotted lines on the left of each panel. They are not true asymptotes because, even for 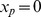, the Legge-Foley transducer is not exactly equal to a power function over a finite range of inputs. The horizontal, dashed lines indicate 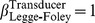. The vertical dashed lines indicate the value of 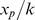 corresponding to the point of inflection of the Legge-Foley transducer. An expression for this quantity is derived in Appendix S2. For typical values of 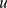 and 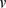, including those in this figure, the point of inflection occurs very close to an input of 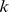, giving 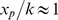. For pedestals above this value, both the target and pedestal will lie in the compressive region of the Legge-Foley transducer, so 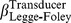 must be less than 1. For this reason, none of the curves enter the top-right quadrant in any of the panels.

For nonzero pedestals, we can substitute 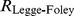 for 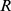 in Equation (17) to obtain, after some work,
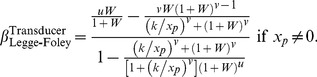
(74)
Figure 13 plots 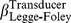 as defined in Equation (74) as a function of 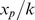, for typical ranges of 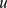, 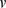, and 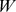. The middle panel of the left column (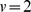, 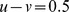) is very close to Legge and Foley's [30] parameters, while the middle panel of the middle column (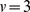, 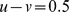) is very close to Meese et al.'s [4] fitted parameters, which we describe in detail later.

**Figure 14:**
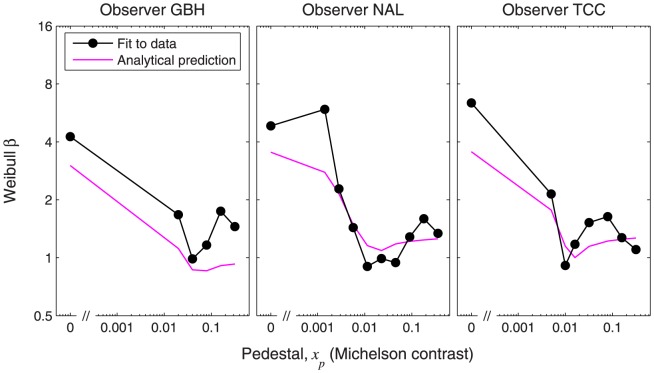
Dipper functions for Weibull 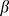 from Henning and Wichmann's data. Weibull 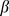 was fitted to Henning and Wichmann's [40] published data as described in the legend of Table 1. These 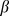 values are plotted in black lines and symbols, excluding observer GBH's 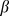 value of 13.1 for a pedestal contrast of 0.01, which is obviously an outlier. In each case, the function mapping pedestal contrast to 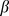 has a dipper shape. To see whether the dip occurred in the predicted location, we fitted a Legge-Foley transducer model to Henning and Wichmann's data separately for each observer. The model's predicted proportion correct, 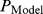, was given by Equation (6) with the transducer function, 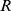, given by the 4-parameter Legge-Foley transducer (Equation (72)), and the noise CDF, 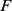, given by the generalized Gaussian (Equation (41)), which had 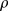 as a free parameter, and 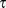 set so that 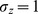, using Equation (44) (thus we adjusted sensitivity by adjusting the transducer gain, rather than the noise CDF spread). For each pedestal value, Henning and Wichmann reported the contrast differences, 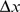, corresponding to three different performance levels (proportion correct, 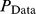 = 0.6, 0.75, or 0.9), sampled from their fitted psychometric functions. We performed a maximum-likelihood fit of the Legge-Foley transducer model to the data, by adjusting the parameters to maximize the likelihood, 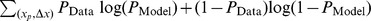. Fitted model parameter sets (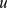, 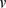, 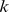, 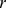, 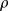) were (3.78, 3.38, 0.0322, 15.3, 0.947) for GBH, (3.36, 3.02, 0.00968, 22.7, 2.09) for NAL, and (3.93, 3.51, 0.0102, 18.9, 2.15) for TCC. For each observer and pedestal value, we used a numerical search method to find the threshold, 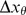, corresponding to a proportion correct of 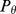, and then calculated the Weber fraction, 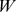, using Equation (34). We then found 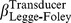 using Equation (74), and 
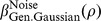
 using Equation (50). The analytical prediction of Weibull 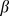 is then given by 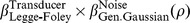, and this is plotted in magenta in the figure. Each observer's global minimum in Weibull 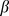 was close to that in the analytical prediction.

One striking feature of these functions is that they all have a dipper shape – as the pedestal increases, 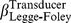 dips down to a minimum and then increases slightly before approaching its asymptote on the right. While it is well known that the discrimination threshold, Weibull 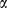, traces out a dipper function as 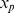 increases from zero [4], [6], [30], [34], [39], to the best of our knowledge no one has ever reported a dipper function for Weibull 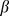 before, so we set out to see if there was evidence for one in the previous literature. We found such a dipper function for 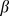 in the data of Henning and Wichmann [40] (see Figure 14 and Table 1). Henning and Wichmann did not report Weibull 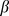, but they reported all thresholds at three different performance levels, allowing us to fit Weibull functions to their data.

For the log and power-function transducers, 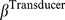 is a function with one or two arguments, so it was practical to test the accuracy of the expressions for a range of plausible arguments. In contrast, 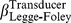 has five arguments, corresponding to three of the transducer parameters, as well as the pedestal and either the Weber fraction or the threshold. To constrain the argument space so that we can test the accuracy of Equations (73) and (74), it is helpful to use values for these arguments that have occurred in real experiments. Legge and Foley's study is not suitable for this because their model had several channels, and did not have the straightforward relationship between stimulus and probability of a correct response described by Equation (6). However, the psychometric function generated by Meese et al.'s [4] preferred model really is a parameterization of Equation (6), so we can assess the accuracy of Equations (73) and (74) for their stimulus values and transducer parameters.

**Figure 15:**
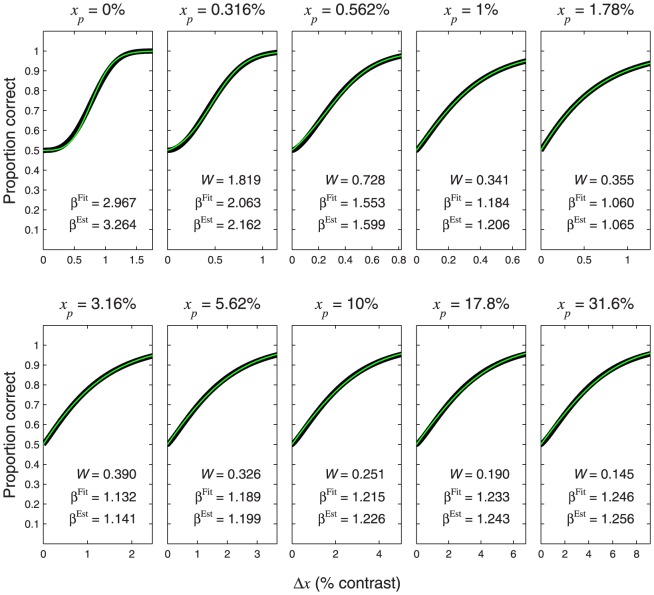
Psychometric functions resulting from a Legge-Foley transducer in Meese et al.'s binocular condition. The thin, green curves show the psychometric functions generated by Meese et al.'s [4] twin-summation model in their binocular condition; in this condition, their transducer is equivalent to the Legge-Foley transducer of Equation (72) with 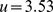, 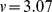, 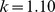, 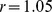. Note 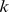 is in units of % contrast, as used by Meese et al.; to convert to units of Michelson contrast, 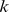 should be divided by 100. The CDF of the noise on the internal difference signal, 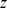, is a cumulative Gaussian with standard deviation given by 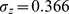. Each panel gives the model's psychometric function for a different pedestal contrast, 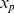, in Meese et al.'s binocular condition. The thick, black curves show the best-fitting (maximum-likelihood) Weibull functions. Each panel displays the 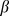 value of the best-fitting Weibull function (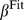) and the estimate, 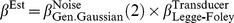, where 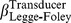 is given by Equation (73) for 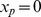, and by Equation (74) for the other pedestal levels. The Weber fraction, 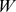, in these equations was calculated from the model's threshold, found by inverting the model's psychometric function using a numerical search method, as explained in the text. Because this model fitted well to Meese et al.'s data, these Weber fractions are close to (but not exactly equal to) the actual Weber fractions obtained in the experiment, given in Table 1.

**Figure 16:**
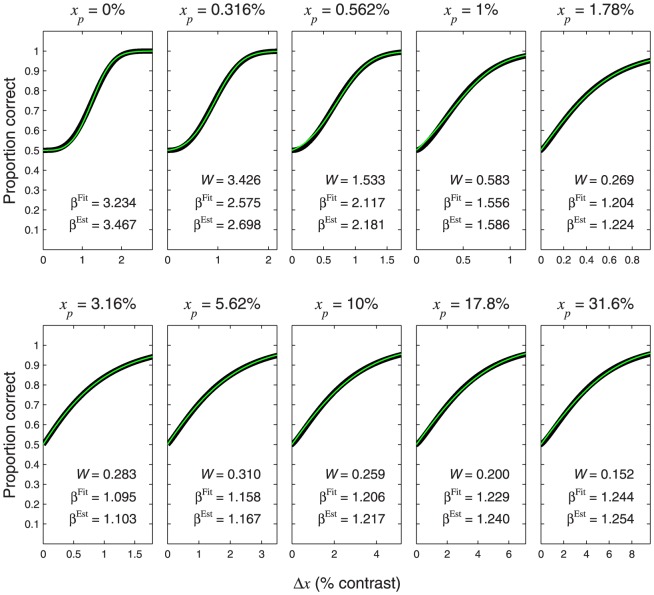
Psychometric functions resulting from a Legge-Foley transducer in Meese et al.'s monocular condition. The same as Figure 15, but for Meese et al.'s monocular condition. In this condition, their twin summation model is equivalent to the Legge-Foley transducer of Equation (72) with the same parameters as those given in the legend to Figure 15, except with 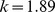 and 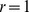.

Meese et al.'s study was on binocular integration, and their data were best fit by a model that they called the “twin summation” model. This model has a transducer that extends Legge and Foley's transducer so that it can handle inputs from left and right eyes:
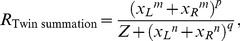
(75)where 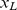 is the stimulus contrast in the left eye, and 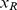 is the contrast in the right eye. In Equation (75), we use upper-case 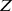 in place of the lower-case 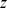 that Meese et al. used, to avoid confusion with our own 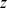, defined in Equation (3). In Meese et al.'s fully binocular condition (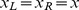), Equation (75) reduces to the standard Legge-Foley transducer of Equation (72), with
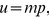
(76)

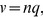
(77)

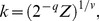
(78)

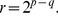
(79)In Meese et al.'s fully monocular condition (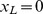 and 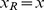 or vice-versa), Equation (75) reduces to the Legge-Foley transducer with 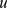 and 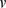 defined as in Equations (76) and (77), but with the other parameters given by
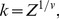
(80)

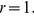
(81)The fitted values of 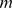, 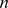, 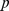, 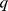, and 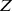 appear in the bottom line of Meese et al.'s Table 2. Using these values, we can specify the equivalent Legge-Foley transducer in the binocular or monocular conditions using Equation (72) with 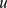 and 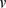 given by Equations (76) and (77), and 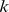 and 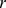 given by Equations (78) and (79) for the binocular condition, and by Equations (80) and (81) in the monocular condition. The values of 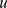, 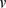, 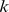 and 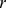 are given in the legend to our Figure 15 for the binocular condition, and Figure 16 for the monocular condition. In summary, although Meese et al. fitted a single transducer function across all their conditions, the equivalent Legge-Foley transducer differs between the binocular and monocular conditions. In both cases, 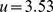 and 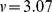, giving 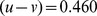, so, although the effective exponent at low contrasts was substantially higher than that of Legge and Foley [30], the effective exponent at high contrasts was similar.

Meese et al. had one further parameter, the standard deviation, 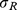, of the noise on the transducer output, which took a fitted value of 0.259. Meese et al. assumed uncorrelated zero-mean Gaussian noise on each signal, so the two sources of noise on each trial would combine to produce Gaussian noise on the internal difference signal with standard deviation given by 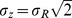. We can therefore define the noise CDF, 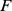, as the cumulative Gaussian with standard deviation 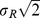. This is equivalent to Equation (45) with 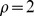 and 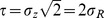.

After we have defined the transducer, 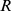, and the noise CDF, 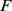, the psychometric function (i.e., the mapping from 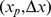 onto probability correct, 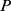) is fully defined by Equation (6). For the Legge-Foley transducer, the psychometric function cannot be inverted algebraically, but the fitted model's threshold, 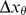, can be found by searching for the contrast difference, 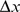, that gives rise to a probability correct of 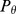. The Weber fraction, 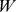, is then given by Equation (34), and the obtained values of 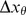 and 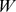 can be used in Equations (73) or (74) (depending on whether or not 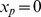), along with 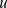, 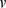, and 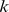, to calculate a value for 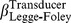 for each pedestal level.

Each panel of Figure 15 shows (in green) the psychometric function generated by Meese et al's twin summation model for a particular pedestal level in their binocular condition. The thick, black curves show the best-fitting Weibull functions. Each panel also compares 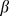 of the best-fitting Weibull function (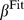) with the estimate, 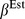, given by 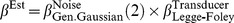, with 
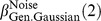
 given by Equation (53) (the model's Weber fractions, 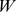, which are used to calculate 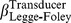, are given in the individual panels). The Weibull fits are good, and the agreement between 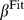 and 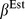 is mostly excellent. Note how Weibull 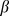 begins to deviate substantially from the linear value (1.3) as the pedestal drops to very low levels, as explained at the end of the Discussion of Theorem 3. Note, too, how both 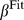 and 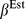 show a dipper function, with the lowest value falling at a pedestal level just above 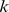 (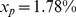); the finding of a dipper function for 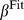 in close agreement with that of 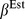 verifies that the predicted dipper function for 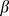 shown in Figures 13 and 14 is a real prediction of the Legge-Foley transducer, and not just a peculiarity of our analytical approximation. Unlike the data of Henning and Wichmann [40], Meese et al.'s [4] data (given in Table 1) do not actually show a dipper for 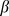, but this is not a serious concern because, as mentioned earlier, Weibull 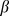 is difficult to measure accurately, and such a small effect on 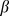 could easily be lost in the experimental noise. Figure 16 shows the same analysis for Meese et al's monocular condition.

## Theorem 4. For Nonzero Pedestals, 2AFC Performance for a Power-Function Transducer Approaches That for a Log Transducer as the Exponent Approaches Zero

### Introduction

We stated earlier that, for a nonzero pedestal, as the exponent of a power function transducer approaches zero, 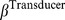 approaches that for a logarithmic transducer, whatever the Weber fraction. This seems a remarkable finding, because the expressions for 
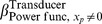
 and 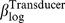 (given by Equations (67) and (70), respectively) appear quite different. In fact, for a given threshold level, the whole 2AFC psychometric function for the power-function transducer and nonzero pedestal (given by Equation (69)) approaches that for a log transducer (Equation (71)) as the exponent, 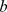, in Equation (69) approaches zero.

Both of these results stem from a more fundamental result: As the exponent, 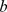, of a power function approaches zero, the function converges towards a log function plus a constant. Because 2AFC performance in the transducer model is based on the *difference* of transducer outputs, this constant cancels out, and, in the limit as 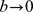, the difference of power functions equals the difference of log functions.

This can be understood from Lemma 1, below.

#### Lemma 1



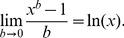
(82)


*Proof*. To begin with, note that, for 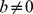,
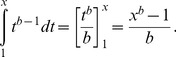
(83)Therefore,
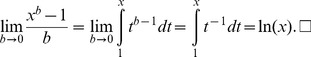



From Lemma 1, we can see that, when 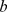 is small,
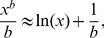
(84)which explains why the power function converges towards a log function plus a constant, 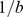, as the exponent, 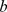, approaches zero. When the transducer outputs are subtracted to make the decision in a 2AFC task, this constant cancels out, so we have
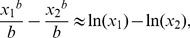
(85)and this is why 2AFC performance for the power function approaches that for a log function as the transducer exponent approaches 0. This does not apply to a zero pedestal because the log function is undefined for zero input.

For those readers who find this informal argument unconvincing, we now prove directly that the two psychometric functions are identical (Theorem 4A) and that the 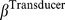 values in the two cases are also identical (Theorem 4B).

### Theorem 4A

#### Statement of Theorem 4A


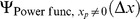
, defined in Equation (69), approaches 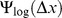, defined in Equation (71), as the power-function exponent, 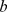, in Equation (69) tends to zero.

#### Proof

From Equation (69),
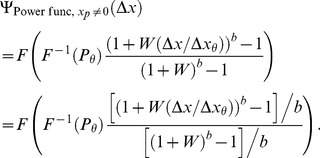
(86)So,
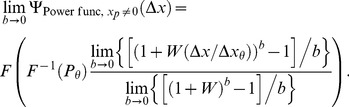
(87)Applying Lemma 1 to the numerator and denominator,
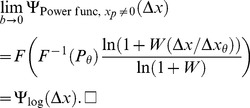
(88)


### Theorem 4B

#### Statement of Theorem 4B

As 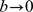, 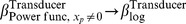.

#### Proof

We can rewrite Equation (67) as
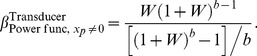
(89)The limit of the numerator of Equation (89) as 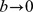 is simply 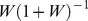, and the limit of the denominator as 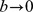 is given by Lemma 1, so we have
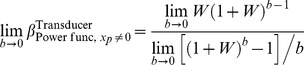
(90)

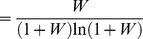
(91)

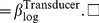
(92)


### Discussion of Theorem 4

**Figure 17:**
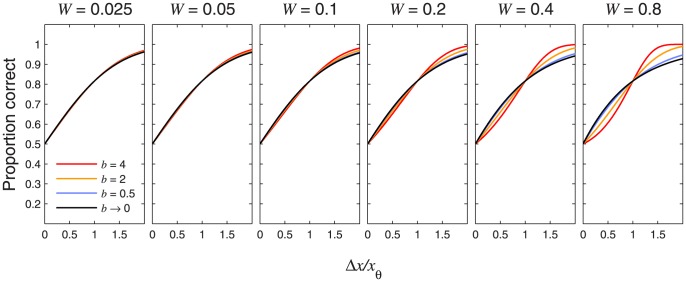
Psychometric functions for the power-function transducer with nonzero pedestal. The psychometric function for 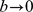 was generated using Equation (71); the others were generated using Equation (69). In both cases, we assumed Gaussian internal noise (i.e. 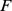 is the cumulative Gaussian). All the psychometric functions go through the point 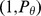, by definition of the threshold (the abscissa is in threshold units, i.e. 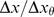). Each panel shows psychometric functions for a particular Weber fraction. Each curve within a panel shows the psychometric function for a particular transducer exponent, 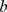. The orange curve (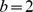) is the psychometric function plotted in green in the second-to-top row of Figure 10. The blue curve (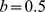) is the psychometric function plotted in green in the second-to-top row of Figure 11. The black line shows the limit as 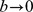. As proved in Theorem 4A, this limiting case is identical to the psychometric function for a log transducer. This is the psychometric function plotted in green in the second-to-top row of Figure 12. This figure illustrates two effects. Within each panel, we see how the psychometric function for the power-function transducer converges towards that for a log transducer as the exponent decreases (Theorem 4). Across panels (right-to-left), we see a demonstration of the effect proved in Theorem 3, whereby, with a nonzero pedestal, all psychometric functions converge towards that for a linear transducer as the discrimination threshold decreases (in this case, since we are plotting psychometric functions for Gaussian noise, the functions converge towards the pure noise CDF of Figure 4B as the Weber fraction decreases).

Theorem 4 shows that, for nonzero pedestals, 2AFC performance with a power function transducer approaches that of a log transducer as the power-function exponent approaches zero. As noted earlier, contrast discrimination data have previously been fit with the Legge-Foley transducer of Equation (72) with parameters set so that, at high contrasts, the transducer was approximately a power function with an exponent of around 0.4–0.5 [4], [30]. Figure 17 shows that an exponent of 0.5 is close enough to zero to make the psychometric function very similar to that from a log function (compare the blue and black curves in Figure 17).

## General Discussion

In 2AFC discrimination experiments, the observer can be modelled using a transducer, followed by constant, additive noise. In this paradigm, the psychometric function is given by Equation (6), where 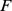 is the CDF of the internal noise, and 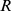 is the transducer function. The model's sensitivity to a stimulus difference, which determines its threshold, can be adjusted by setting the gain on the transducer (i.e. stretching or compressing 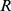 vertically) while keeping the spread of the noise CDF constant at some convenient level, or by setting the spread of the noise CDF (i.e. stretching or compressing 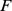 horizontally) while keeping the gain of the transducer constant at some convenient level. Theorem 1 reformulates Equation (6) so that both the transducer gain and the spread of the noise CDF can be set to convenient levels, and the threshold can be set directly.

Although the presentation of the theorems in this paper makes heavy use of the theoretical framework in which the stimulus signal is put through a transducer, and stimulus-independent noise is added, it is not necessary to accept this model to find the theorems useful: All we need to assume is that the psychometric function has a form *consistent* with such a model. For example, the intrinsic uncertainty model contains no transducer, but generates a psychometric function that closely approximates that of a power-function transducer with additive Gaussian noise [21], so the theorems in this paper can be applied to that model as if was a transducer model.

Nevertheless, the theorems do have added value if we go along with the transducer model, because they give an insight into the roles played by the different elements of the transducer model in determining the form of the psychometric function. In the next section, we give a summary of some of the insights that we have gained into the Weibull function. The sections after that examine some of the issues in more detail; each of these detailed sections is self-contained, and any of them can be skipped without affecting the intelligibility of the other sections.

### The Weibull Function

Functions of proportion correct against stimulus difference are often fitted with a Weibull function, which has two parameters of interest: the threshold, 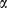, and “slope” or “shape” parameter, 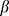. Most psychophysical research has focussed on the threshold, but 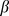 can be informative too, and has proved useful when competing models make quite similar predictions of threshold [4]. Theorem 2 shows what happens to 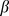 when the Weibull function is fitted to the psychometric function for the transducer model, given in Equation (6). This theorem shows that 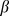 can be partitioned into two factors: 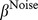, which depends only on the shape of the internal noise distribution, and 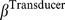, which depends on the transducer function, and can also depend on the pedestal level and the observer's threshold. Weibull 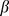 is estimated by multiplying these two factors together. 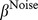 is the estimate of the 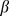 of the Weibull function that fits best to the noise CDF. We found that, for all the noise CDFs in Figure 7, 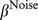 accurately estimates the best-fitting Weibull 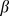, which validates the accuracy of our general expression for 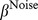 (Equation (16)) for a range of noise CDFs. From our general expressions for 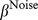 and 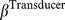, we derived expressions for several specific cases. In each case, these specific expressions provided accurate estimates of the fitted Weibull 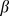
s, and will do so in any other situation in which we can express the observer's or model's true psychometric function in the form of Equation (6), and the Weibull function provides a good fit (this is because the only premise of Theorem 2 is that the Weibull function can be adjusted to provide a good fit to Equation (6)).

As well as providing a convenient formula to estimate Weibull 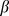, our theorems give many insights into the genesis of this parameter. By partitioning the expression for 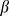 into the two factors, 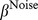 and 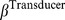, we can understand the separate contributions made by the noise distribution and the transducer.

One insight is that Pelli's [19] finding (that 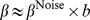 for a power-function transducer and zero pedestal) is a specific instance of the more general expression (Equation (15)) that we derived in Theorem 2. In our terms, the “
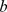
” part of Pelli's relation is 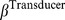, and our general expression for 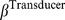 reduces to 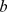 for a power function transducer and zero pedestal.

Another insight relates to 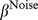: Since 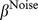 is a number that depends on the noise distribution, changing the noise distribution simply changes all the Weibull 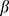
s by a fixed proportion. For example, we showed that 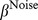 for Gaussian noise is larger than 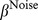 for Laplacian noise by a factor 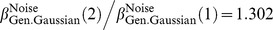 (see Equations (52) and (53)); therefore, changing from Laplacian to Gaussian noise without any other change will increase Weibull 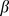 in every situation by a factor 1.302.

A further insight relates to 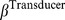: Theorem 3 proves that, as long as the gradient of the transducer function is not 0 or 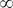 at the pedestal level, 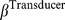 approaches 1 as the discrimination threshold decreases. We showed that the Weber fractions generally obtained for contrast discrimination between two easily visible stimuli are small enough to make 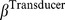 close to 1, the value for a linear transducer. Therefore, in this case, Weibull 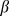 is close to 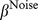, which is about 1.3 for Gaussian noise. Since the Central Limit Theorem provides a good reason for assuming that the noise should be approximately Gaussian, this explains why Weibull 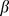 turns out to be close to 1.3 for suprathreshold contrast discrimination (although, as explained below, in the section headed “The shape of the internal noise distribution”, the fitted 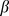 values in Table 1 are in general slightly too high to be consistent with a Gaussian, suggesting a distribution with lower kurtosis).

The linearizing effects of the pedestal have been noted before [4]. Given that all differentiable functions are “locally linear”, one might argue that the surprising thing is not that performance becomes linear with decreasing discrimination threshold, but that there are cases where this does not happen. A commonly encountered example of the latter is the case of a power-function transducer and zero pedestal, analysed previously by Pelli [19]. Here, 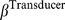 is always equal to the power-function exponent, so psychophysical performance never becomes linear, however small the threshold gets. It is not that the power function disobeys the rule that all differentiable functions are locally linear, but rather that the definition of local linearity used in the definition of a differentiable function is too weak for our purposes. In the next section, we introduce a different definition of local linearity that is strong enough to determine whether or not linear behaviour will emerge as the discrimination threshold decreases. We show that this “strong local linearity” is not shown by the power function at 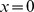.

### “Local linearity” and Weibull *β*

**Figure 18:**
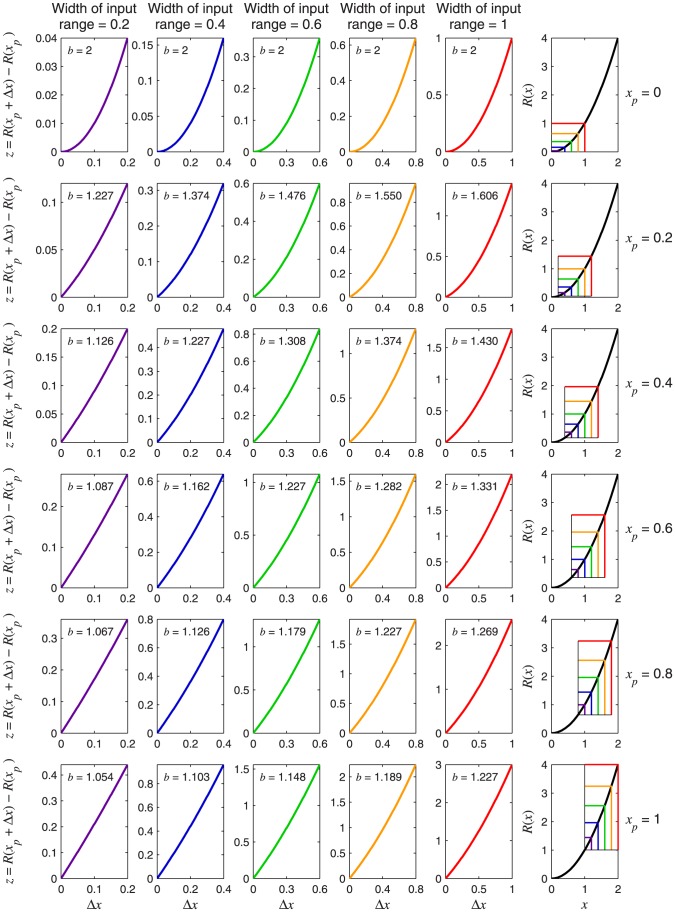
Effect of a pedestal on the linearity of an expansive power-function transducer. Each panel in the rightmost column shows the same expansive power-function transducer given by 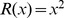. The panels to the left show parts of this transducer sampled over different ranges of inputs: The width of the range is varied across columns of panels, and the lower limit of the range is varied across rows of panels. The lower limit would correspond to the pedestal value, 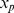, in a discrimination experiment. The abscissa of the curves on the left is the stimulus difference, 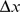, and the ordinate is 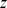, the difference in internal signal values after transduction. The 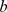-value given in each panel is the exponent of the power function that fits best (least squares) to these curves. Each coloured box drawn on a transducer in the right column indicates the part of the transducer that is sampled by the correspondingly coloured curve given in a panel to the left on the same row. It can be seen that, as the pedestal increases, the best-fitting exponent quickly approaches 1, giving an approximately linear mapping from 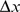 to 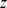. This linearizing effect is enhanced as the width of the range decreases. 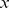, 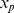, and 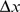 are given in arbitrary units: For a given transducer, the best-fitting exponent is determined by the *ratio* of the pedestal value to the width of the input range. For example, with the transducer shown here, when the pedestal value is equal to the width of the range, the best-fitting exponent is always 1.227; when the pedestal is twice the width of the range, the best-fitting exponent is always 1.126. For a zero pedestal (top row), the best-fitting exponent is always 2, regardless of the width of the input range, and in this sense the power function is not “strongly locally linear” at 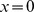.

One might think that the tendency towards linear behaviour with decreasing discrimination threshold is just a trivial consequence of the fact that any differentiable function is “locally linear”: The definition of differentiability requires that a function be “well approximated” by a linear function near the point of interest. However, the definition of “locally linear” that appears in the test of differentiability is not sufficient to guarantee linear psychophysical discrimination behaviour for small thresholds. As we saw earlier, for a power-function transducer, 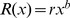, and zero pedestal, 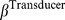 is always equal to 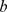, however small the threshold gets. Theorem 3 does not apply in this case (except when 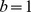), because, when 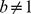, the gradient of the transducer is 0 or 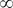 at a pedestal level of zero. For 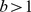, the power function is differentiable at 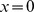, and so it is locally linear in the sense required by the definition of differentiability, but it does not generate linear behaviour for small thresholds. We can see this in Figure 18, which shows an expansive power-function transducer with exponent 2. As the pedestal value increases from zero, the function mapping the stimulus difference, 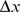, onto the internal difference signal, 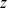, appears increasingly linear, and this linearizing effect becomes more pronounced as the range of inputs decreases. But, when the pedestal value is zero, the mapping from 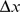 to 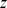 always has the same form as the transducer, 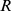, regardless of the range of inputs. However much we zoom into the power function at 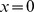, it still looks like a power function with the same exponent. So there is clearly a sense in which an expansive power function is *not* locally linear at 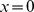. What is going on?

To make sense of this, we need to consider exactly what we mean when we say that a differentiable function must be locally linear. What follows is equivalent to the definition of differentiability given by Hasselblatt and Katok (Ref. [41], p. 400), but simplified to deal with functions of one variable only. For a function, 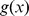, to be differentiable at 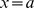, there must be some straight line, 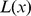, through the point 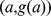, such that 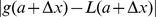 approaches zero more quickly than 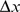 does. More formally, 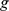 is differentiable at 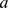 if and only if there exists a number 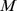 such that, if we define 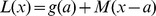, then
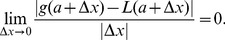
(93)If this condition is satisfied, then 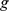 is differentiable at 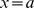, and 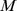 is the derivative of 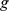 at that point. An expansive power function clearly satisfies this condition for 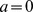. In this case, 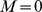 and 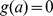, so 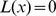 for all 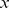, and 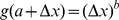. Thus,
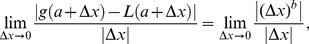
(94)and, for 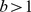, the limit in Equation (94) is zero.

**Figure 19:**
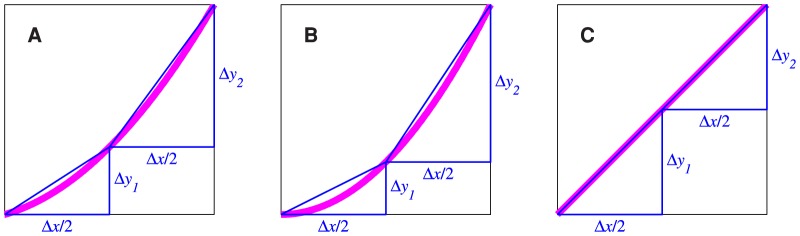
Index of acceleration, 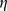. (A) The wide, magenta curve shows an expansive power function sampled over a range of inputs from 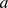 to 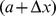, where 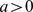. The horizontal blue lines both have length 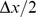, and the vertical blue lines have length 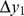 and 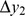 as indicated. The slope, 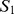, of the secant (the oblique line) across the left half of the curve is given by 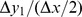, and the slope, 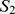, of the secant across the right half of the curve is given by 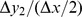. Our index of acceleration, 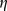, is given by 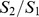. For the power function, when 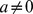, 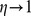 as 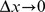, so the curve is “strongly locally linear” at 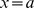. (B) The same as A, but with the bottom of the range of inputs, 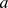, equal to zero. In this case, 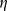 depends only on the exponent of the power function, and so it does not approach 1 as 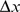 approaches zero. The power function is not “strongly locally linear” at 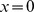. (C) The same as A, but for a straight line function. Here, 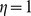 for all 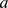 and 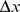.

So the expansive power function is locally linear at 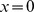 in the sense required for differentiability. However, when we look at the top row of Figure 18, we can see that it will never look like a straight line, however much we zoom in. To capture this behaviour, we need a different definition of “locally linear”, and the key property of linear functions that we need to appeal to is the fact that the gradient of a linear function is constant. For any function, 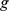, Let 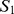 be the slope of the secant between the points 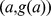 and 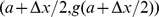, and let 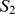 be the slope of the secant between the points 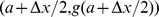 and 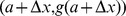. Figure 19 illustrates these secants for three types of function over the range 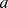 to 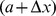: an expansive power function where 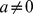 (Figure 19A), an expansive power function where 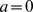 (Figure 19B), and a straight line (Figure 19C). The slopes, 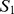 and 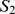, of these secants are given by
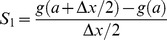
(95)and
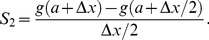
(96)We define the curve's “index of acceleration”, 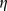, as
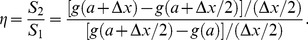
(97)For an expansive function, the slope increases towards the right, so 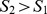, and 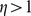; for a compressive function, 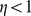; and, for a linear (or, strictly speaking, affine) function, 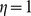. We can therefore take the limit of 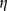 as 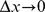 to indicate whether the function is *locally* expansive, compressive, or linear at 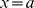. We classify a function as being “strongly locally linear” at 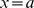 if 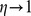 as 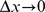. This precisely captures the kind of local linearity that is relevant to Weibull 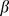. In general, the numerator and denominator on the right hand side of Equation (97) both approach the derivative, 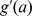, as 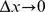, and so, as long as the gradient of 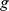 at 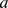 is not 0 or 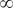, we have
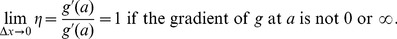
(98)Thus, all differentiable functions are “strongly locally linear” at 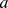 except those with zero gradient at 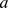 (those with infinite gradient at 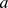 are not differentiable at 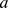 anyway, and are not locally linear by either definition). If the gradient at 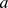 is zero, then Equation (98) gives us the indeterminate form 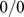, so the limit of 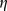 cannot be evaluated using Equation (98), and the function will not necessarily be strongly locally linear at 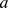. The conditions necessary for Equation (98) to apply are the premises of Theorem 3. Thus, we can now see what is happening in Theorem 3. In all cases for which the premises of Theorem 3 are satisfied, the transducer function is strongly locally linear at the pedestal level, and so linear behaviour will be expected to emerge as the threshold decreases, and we sample a progressively smaller range of inputs. For the case of a power function, 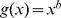, with 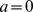 (Figure 19B), we can go back to Equation (97) to derive the limit of 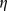. In this case, we have
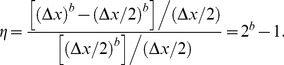
(99)Thus, the index of acceleration is 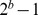, whatever the value of 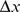. The limit of 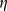 as 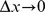 is therefore not 1 (unless 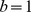), and so the power function is not strongly locally linear at 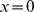. The ratio of the slopes, 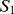 and 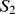, of the secants is unchanged as 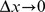, and so the shape of the power function does not become any more linear as we zoom in, as shown in Figure 18.

In summary, the definition of local linearity embodied in the definition of a differentiable function is not strong enough to explain why discrimination performance does not always approach that for a linear transducer as the discrimination threshold decreases. We introduced a different definition of local linearity, which we call “strong local linearity”, and it is only when the transducer conforms to this stronger definition of local linearity at the pedestal level that we should start to see linear behaviour as the discrimination threshold decreases.

### Relationship between Weibull *β* and log-log slope of *d*′ against stimulus level

As mentioned earlier, in a detection task (i.e. where the pedestal is zero), if 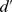 is a power function of stimulus level (with exponent 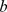), then the resulting psychometric function is given by Equation (9), which has the same form as Equation (6) with a power-function transducer and Gaussian noise. In this scenario, if 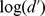 is plotted against 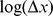, the resulting function is a straight line with slope 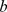. Pelli [21] was the first to appreciate the relationship between 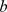 and Weibull 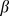, showing that, for the intrinsic uncertainty model,
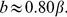
(100)He later realised that this relationship is not specific to the uncertainty model, but instead applies to *any* model for which 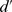 is a power function of stimulus level [19]; the intrinsic uncertainty model shows this relationship because 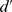 is approximately a power function of stimulus level in this model.

In his earlier paper, Pelli [21] derived Relation (100) from the uncertainty model, for which the psychometric function does not fit perfectly to either the Weibull function, or Equation (9) (for which 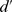 is a power function of stimulus level). In his later paper [19], he assumed a model for which the psychometric function was precisely that of Equation (9), and found the best-fitting Weibull function. This resulted in the relationship, 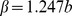, which can be inverted to give 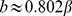, which is the same as Relation (100) within the specified margin of error. So, in Pelli's earlier analysis [21], using the uncertainty model, both the Weibull function and Equation (9) were approximations, whereas his later analysis [19] assumed Equation (9) to be precisely correct, and the Weibull function to be an approximation. Strasburger [29] took the one remaining option, which is to assume that the Weibull function is precisely correct, and Equation (9) is an approximation. For several different Weibull functions (with different 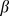 values), he plotted 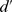 against stimulus level. Because, in Strasburger's analysis, Equation (9) was an approximation, the log-log plots of 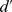 against stimulus level were not exactly straight lines, but they were nearly straight for 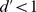. Strasburger found the change in log stimulus level between 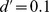 and 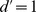, and used this to define the slope, and this resulted in a similar relationship to that of Pelli, but with a slightly higher constant of proportionality: 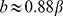.

Our equations give an alternative approach to formulating this relationship. If we assume, like Pelli [19], that Equation (9) is precisely correct, then the “true” psychometric function is identical to that from a power-function transducer, zero pedestal, and additive Gaussian noise. In this case, 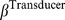 is given by Equation (63), and 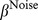 is given by Equation (53), giving 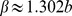, or 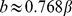. The reason why our equations yield a lower constant of proportionality than Strasburger's is that our expressions for Weibull 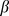 are based on the psychometric function at the performance level 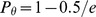. From Equation (8), this corresponds to a 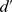 level of 1.27, where Strasburger's log-log plots of 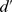 against stimulus level start to become noticeably shallower. Strasburger's slopes were derived between 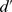 levels of 0.1 and 1, which correspond to performance levels of 0.53 and 0.76, respectively, i.e. approximately the bottom half of the psychometric function; if we fitted the Weibull function to the bottom half of the true psychometric function, we should expect to get a different value for 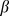 than if we fitted across a wide range of performance levels.

So far, we have focussed on the relationship between Weibull 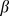 and the exponent of a simple power function. Klein [36] examined the relationship between Weibull 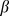 and the exponent of the numerator of the Legge-Foley transducer (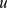 in Equation (72)). He expressed the Legge-Foley transducer slightly differently from Equation (72):
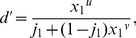
(101)where 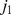 is a constant, and 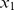 is the stimulus level, 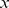, divided by the stimulus level that gives 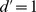. The subscript, “1”, on 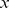 and 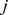 in Equation (101) indicates the value of 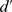 that we obtain when 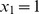; it is easily seen that, if 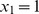 in Equation (101), then 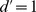, giving a performance level of 0.76. Equation (101) can produce log-log plots of 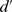 against stimulus level very much like those derived by Strasburger for the Weibull model, becoming more shallow with increasing stimulus level. This suggests that the model described in Equation (101) is a better approximation of the Weibull model than the simple power-function transducer.

To find the psychometric function for Klein's model (defined in Equation (101)), we can use Equation (101) to substitute for 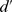 in Equation (8), and obtain
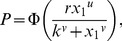
(102)where
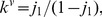
(103)and
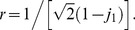
(104)
Equation (102) is the psychometric function that would arise from a Legge-Foley transducer with zero pedestal, and additive, unit-variance, Gaussian noise on the internal difference signal, 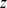.

Klein constrained the transducer parameters so that 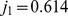, and 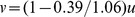. Thus, the only free parameter of the model was 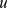. He found that, for any 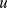, and any stimulus level, 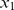, the psychometric function defined by Equation (102) was extremely close to a Weibull function with 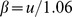. Klein remarked that he was very surprised to discover that this fixed relationship between 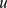 and 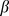 held for all values of 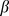 without having to change the other parameters of the Legge-Foley transducer. But we can explain this surprising finding by using our expression for 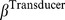 for the Legge-Foley transducer and zero pedestal (Equation (73)). First, let 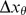 be the threshold value of 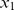 corresponding to a performance level of 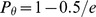. For any value of 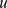, 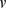 is determined by 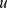, and both 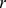 and 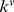 are determined by 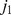, and we can find 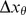 by numerical search; we can then plug these values of 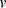, 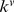 and 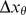 into Equation (73) to obtain an expression for 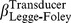 in terms of 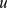. When we do this, we always obtain 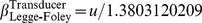 (to 10 decimal places). We estimate Weibull 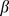 by multiplying 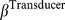 by 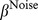, and as already noted, the model defined in Equation (101) implies Gaussian noise, so 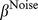 is given by Equation (53). When Equation (53) is evaluated to 14 decimal places, we find 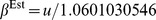 (to 10 decimal places) for any 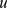, supporting what Klein found.

To understand why 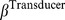 is a fixed multiple of 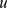, we need to express the stimulus in different units. Let 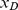 denote the stimulus level, 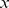, divided by the stimulus level that gives 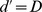, where
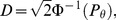
(105)with 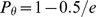. We can then define the 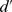 function as
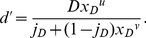
(106)
Equation (106) has the same form as Equation (101), but with the stimulus expressed in different units. When 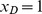, Equations (106) and (105) give 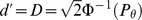, and so, from Equation (8), the proportion correct is 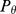. Thus, when we express the stimulus in units such that the stimulus level is 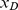, the threshold value, 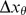 (which we have defined to be the stimulus value corresponding to a performance level of 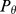), is simply given by
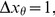
(107)rather than having to be found by numerical search.

Using Equation (106) to substitute for 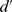 in Equation (8), we obtain
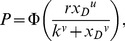
(108)where
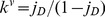
(109)and
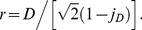
(110)Let us again assume that 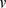 is some fixed multiple, 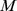, of 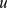, as it is in Klein's example:
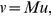
(111)for constant 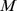. Using Equations (107), (109) and (111) to substitute for the terms in Equation (73), and simplifying, we find
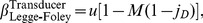
(112)and so, for constant 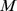 and *j_D_*, 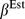 is a fixed multiple of 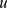, given by
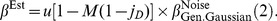
(113)This explains Klein's surprising finding that, with the constraint that 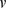 is a fixed multiple of 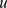, there is a fixed multiplicative relationship between 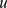 and 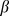 that holds for all values of 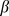 when the other Legge-Foley transducer parameters are held constant.

### The shape of the internal noise distribution

For several decades, the internal noise in psychophysical models has usually been assumed to be Gaussian, but recently, Neri [18] argued that it has a Laplace distribution, which has considerably higher kurtosis than a Gaussian. This conclusion was reached using reverse correlation techniques to investigate detection of bar stimuli embedded in noise.

But do Neri's conclusions about the internal noise also hold for noise-free stimuli? It is not possible to use Neri's methods to study the internal noise when the stimuli are noise-free because these methods require substantial amounts of noise to be added to the stimuli. For noise-free stimuli, we can learn something about the internal noise from the 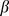 of the fitted Weibull psychometric function, because the shape of the noise distribution affects Weibull 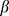 through the factor 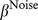 in Equation (15). If we knew the value of 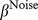, that would greatly narrow down the set of possible internal noise distributions. The key difficulty is that, as noted by Neri [18], the internal noise distribution is confounded with the deterministic transformation, i.e. the transducer. This confound is made explicit in Equation (15), where Weibull 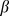 is shown to be the product of 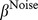 and 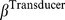. Since psychophysical measurements are generally affected by both the internal noise and the transducer, we are limited in the conclusions that we can draw about the internal noise distribution. For example, a Weibull 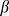 of 1.3 is consistent with Gaussian internal noise and a linear transducer, because 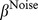 for the Gaussian is 1.3 (Equation (53)), and 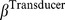 for a linear transducer is 1; but, since 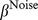 for Laplacian noise is 1 (Equation (52)), a Weibull 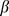 of 1.3 is also consistent with Laplacian internal noise and a combination of transducer, pedestal, and threshold that yields 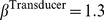. A partial solution to this problem is to focus on experimental situations where it is likely that 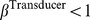; then it follows from Equation (15) that 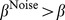. In this case, the fitted Weibull 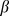 places a lower bound on 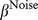, and possible internal noise distributions will be those for which 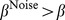.

One situation where we can be reasonably sure that 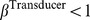 is suprathreshold contrast discrimination. If the internal noise is additive, then the transducer in the suprathreshold region of the contrast axis has to be compressive to account for the rise in discrimination threshold with increasing pedestal for suprathreshold pedestals, as found by numerous researchers [4], [6], [30]–[32], [34], [39], [40], [42]. As explained in the discussion of Theorem 2, and Figure 5, a compressive transducer will give rise to 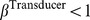. Thus, for suprathreshold contrast discrimination, although we cannot determine the exact value of 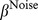 from the psychometric function, we know it must be greater than the fitted Weibull 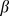. Looking at Table 1, most of the Weibull 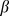 values for suprathreshold contrast discrimination fall above 1, and so 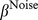 in these cases must be greater than 1, and therefore inconsistent with a Laplace distribution. Out of 38 suprathreshold discrimination conditions (i.e. where the pedestal is greater than the detection threshold), 31 conditions gave a fitted 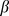 that was greater than 1.

In general, there will be many different pairs of noise distribution and transducer function that are consistent with the data. Suppose we just consider generalized Gaussian noise distributions (parameterized by the shape parameter, 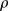) and power-function transducers, parameterized by the exponent, 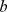; most transducers would usually be well-approximated by a simple power function over the limited range of inputs spanned by the psychometric function. For a given empirically obtained psychometric function, we could then plot a contour of all the possible pairs of 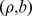 that are consistent with the empirical data. We will now do this for the data in Table 1.

For generalized Gaussian noise, 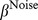 is given by 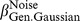 in Equation (50), which is determined by the shape parameter, 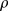. For a power function transducer, 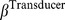 is given by 
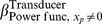
 in Equation (67), which is determined by the transducer exponent, 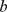, and the Weber fraction, 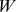. For these forms of noise and transducer, the fitted Weibull 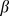, which we call 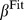, should be related to 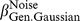 and 
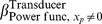
 according to the following approximation:
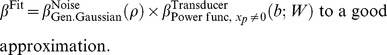
(114)In Equation (114), we explicitly indicate that 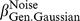 is a function of 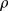, and 
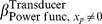
 is a function of 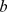 and 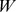. For a given empirically obtained psychometric function, we know the fitted Weibull 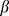, i.e. 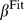, and the fitted Weber fraction, 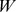 (these values are given in Table 1), and we can plug these values into Equation (114), to give an equation with two unknowns, 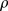 and 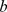. It is not possible to rearrange this equation algebraically to make either 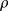 or 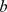 the subject; however, for any 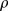, we can search for the 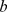 that satisfies the equation. This allows us to trace out a contour of all the possible pairs 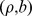 that are consistent with the fitted 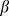 and 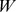.

**Figure 20:**
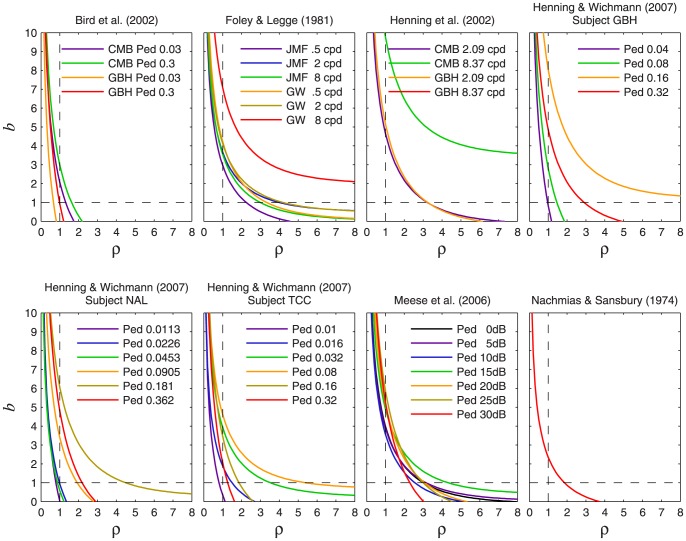
Pairs of noise distribution and transducer exponent consistent with the Weibull parameters for contrast discrimination. 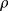
 is the generalized Gaussian CDF shape parameter, and 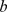 is the power-function transducer exponent. Each curve plots the set of 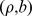 pairs consistent with one of the fitted psychometric functions for suprathreshold contrast discrimination given in Table 1 (non-starred conditions). Where available, we used the fitted 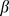 and 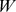 parameters from the Weibull fit that included the lapse rate parameter, 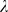. Note that the contour for Henning et al.'s subject GBH in the 8.37 cpd condition lies out of range of the axes in this figure, and so is not visible. This is because the fitted Weibull *β* of 6.70 is much higher than usually found – almost certainly an unreliable measurement.

Figure 20 plots the 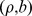 contours for the suprathreshold conditions given in Table 1 (i.e., the non-starred conditions). If we assume the noise is Laplacian, then the transducer exponent consistent with the data can be read off by seeing where the contour for that condition intersects the vertical dashed line (corresponding to 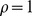). For most conditions, the exponent, 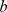, would have to be substantially greater than 1 to be consistent with both the data and the Laplacian assumption. As argued earlier, the transducer for suprathreshold contrast discrimination should be compressive, and would fit best to a power function with 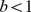, so the range of possible 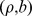 pairs are those that lie below horizontal dashed line (corresponding to 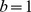). The lowest possible values of 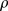 consistent with a compressive transducer are those where the contours intersect the horizontal dashed line. For the conditions in Figure 20 that do intersect the horizontal dashed line, the median point of intersection is given by 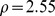, implying a distribution that has *lower* kurtosis than a Gaussian, the opposite of Neri's proposal. Furthermore, note that 2.55 is the median of the *minimum possible* 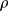 values, corresponding to the limit as the compressive transducer approaches linearity. For a more substantially compressive transducer (i.e. 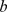 substantially less than 1), the 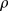 values plotted in Figure 20 are higher, corresponding to distributions with substantially lower kurtosis than a Gaussian.

How can we reconcile these results with those of Neri, which suggest that the noise has *higher* kurtosis than a Gaussian? One possibility is that the shape of the noise distribution is dependent on the stimuli, with noisy stimuli somehow inducing a Laplacian internal noise distribution, while noise-free stimuli induce an internal noise distribution that has much lower kurtosis. Another possibility is that the assumption of additive, stimulus-independent, noise may be incorrect. For example, Kontsevich and Tyler [43] argued that the transducer is an expansive power function (with exponent 2–2.7) over the whole contrast range, and the increase in contrast discrimination threshold with increasing pedestal is caused by an increase in the noise variance with increasing contrast. While the additive and variable noise models are barely distinguishable on the basis of Kontsevich and Tyler's data (see Ref. [44]), the possibility of variable noise has some support from 2-response 4AFC experiments [15], [45], and might resolve the apparent conflict between Neri's results and those in Table 1. If Kontsevich and Tyler are correct that the transducer is an expansive power function across the whole contrast range, then this would result in a higher Weibull 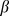 than a compressive function, and in this case, the 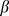 values of around 1.4 obtained for suprathreshold contrast discrimination may well be consistent with Laplacian noise with variance that increases with contrast. Further consideration of this hypothesis falls outside the scope of this paper, because here we are mainly concerned with formal relationships between models and psychometric functions within the theoretical framework of a transducer and additive noise.

### Relationship between power function and log transducers

Theorem 4 showed that, as the exponent of a power function transducer approaches zero, 2AFC behaviour approaches that for a log transducer. This comes about because, in the limit as the exponent tends to zero, the difference of power functions becomes proportional to the difference of logs. This gives us an insight into what determines the difference between the two fitted exponents in the Legge-Foley transducer. Recall that, for high inputs, the Legge-Foley transducer approaches a simple power function with exponent 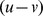. For typical Weber fractions of around 0.3, the transducer exponent makes little difference to the predicted Weibull 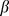 (see Figure 9), so the fitted exponent is more strongly constrained by the threshold, 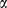, that it predicts for each pedestal level. If threshold is proportional to the pedestal, then we have Weber's law (i.e. the Weber fraction, 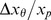 is constant, so that a plot of 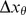 against 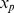 is a straight line with a slope of 1 on log-log axes). A logarithmic transducer would generate Weber's law [46]; this is because, for additive noise, the discrimination threshold, 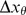, corresponds to a constant internal difference signal, 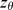, and a logarithmic transducer would give 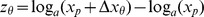, implying 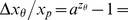 constant, which is Weber's law. On the other hand, a linear transducer would cause 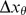 to be constant with respect to 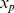, so that the plot of 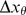 against 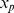 was a straight line with a slope of 0. As the exponent of the power function transducer increases from infinitesimally above zero (giving the same performance as a log transducer) to 1 (giving a linear transducer), the slope of the plot of 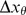 against 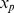 on log-log axes will gradually decrease from 1 to 0. Actual slopes obtained in the literature usually fall between 1 and 0.6 [4], [6], [32], [34], and this would require an exponent between 0 and 1, which explains why the difference between the fitted exponents in the Legge-Foley transducer [4], [30] falls in this range. A corollary of Theorem 4 is that no power function transducer could generate a log-log slope of 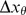 against 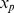 that was greater than 1: As the exponent increases from zero, the slope decreases from 1. This also applies to any transducer that approximates a power function for high inputs, such as the Legge-Foley transducer.

### Lapse rate

As noted earlier, psychophysical observers sometimes respond incorrectly, even on easy trials. This may be due to lapses of concentration, so that the observer either did not look at the stimuli, or cannot remember which interval contained the target; on such trials, the proportion correct will be 0.5. Suppose the proportion correct on non-lapse trials is given by 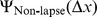. Then, if lapse trials occur with probability 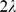, the probability of a correct response overall will be given by 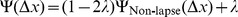. The effect of 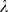 is to linearly compress the psychometric function vertically so that the upper asymptote is 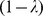. For simplicity, our analytical results regarding Weibull 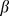 are derived assuming 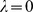, but it is important to realize that these results still apply for non-zero lapse rates. To understand why, note that, if we change the true psychometric function so that the lapse rate is non-zero, the psychometric function will be vertically compressed but otherwise unchanged. Thus, the best-fitting Weibull function will be one that is vertically compressed but otherwise unchanged. This change to the Weibull function is achieved by increasing 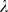 while keeping 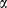 and 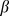 the same, so the 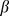 of the best-fitting Weibull function is unchanged by introducing a non-zero lapse rate. Our results about Weibull 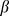 therefore suffer no loss of generality by being derived under the assumption of 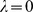.

## Conclusions

We analyzed the psychometric function within the theoretical framework of a transducer and additive noise. We showed that, for a variety of commonly used transducers and noise distributions, the true psychometric function was well fit by a Weibull function. We showed that Weibull 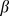, which controls the Weibull function's shape on a linear abscissa, can be partitioned into two factors. One, which we call 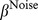, is the 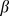 of the Weibull function that fits best to the CDF of the noise on the internal difference signal. The other factor, which we call 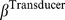, depends on the transducer function and pedestal level, and can also depend on the observer's threshold. To a close approximation, the 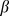 of the Weibull function that fits best to the true psychometric function will be given by 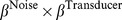. We derived general expressions for 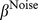 and 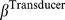, and, from these, derived specific expressions for particular noise distributions and particular transducers. We showed that, for a wide range of noise distributions and transducers, the fitted Weibull 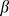 was closely matched by 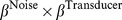. For a power function transducer with exponent 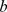, and zero pedestal, 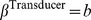, which gives us the relationship between Weibull 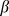 and 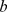 derived by Pelli [19]. The power of our approach is that it can easily be applied to any noise distribution and any transducer, provided that the Weibull function provides a good fit to the psychometric function.

We also explained why, as the discrimination threshold decreases, 2AFC behaviour will approach that for a linear transducer for suprathreshold discrimination, but not for detection. Although most transducer functions are differentiable (and therefore locally linear in one sense), we showed that, at the point at which the gradient of a nonlinear function is zero, the function fails a stronger test of local linearity, and it is this stronger kind of local linearity that is critical for determining whether or not behaviour becomes linear with decreasing threshold. For detection experiments, the transducer usually has zero gradient at the (zero) pedestal level, and is not “strongly locally linear” in the sense that we defined, and this prevents the psychophysical behaviour from approaching that for a linear transducer as the threshold decreases.

In Theorem 4, we showed that, as the exponent of a power function approaches zero, psychophysical behaviour approaches that for a logarithmic transducer. A corollary of this theorem is that the log-log slope of the threshold vs pedestal curve can never exceed 1 for a power-function transducer and additive noise.

Finally, an understanding of the factors that determine Weibull beta gives us some insight into the shape of the noise distribution. In apparent contrast to a recent claim [18] that the internal noise has considerably higher kurtosis than a Gaussian distribution (based on experiments on detection of a bar embedded in noise), our analysis of suprathreshold contrast discrimination with noise-free stimuli suggests that the internal noise does not have higher kurtosis than a Gaussian; if anything, the internal noise appears to have *lower* kurtosis than a Gaussian. Both our analysis and that of Neri [18] made the assumption of additive, stimulus-independent noise, and we suggest that one possible resolution of this apparent contradiction might be to drop that assumption.

## Supporting Information

Appendix S1
**Proof that** 
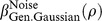
 **asymptotes to** 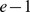 **as** 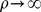 **.**
(PDF)

Appendix S2 **Point of inflection of the Legge-Foley transducer.**
(PDF)
