# Supplementary material for: Four Theorems on the Psychometric Function
Source: PLoS One. 2013 Oct 4;8(10):e74815. doi: 10.1371/journal.pone.0074815 (PMC3790801; doi:10.1371/journal.pone.0074815)
Supplement: Appendix S1 — Proof that asymptotes to as . (PDF) [file pone.0074815.s001.pdf]

Appendix S1. Proof that $\beta^{\text{Noise}}_{\text{Gen.Gaussian}}(\rho)$ asymptotes to *e* – 1 as $\rho \to \infty$

First, note that the generalized Gaussian PDF asymptotes to a rectangular distribution, centred on 0, as $\rho \to \infty$. The width of the distribution depends on τ, but we don't need to know its value, as it cancels out, so let us call the width w. The CDF, $F_{\text{Gen.Gaussian}}(z;\infty,\tau)$ is thus a linear ramp that rises from 0 at $z = -w/2$ to 1 at $z = w/2$. Its gradient at all points over the sloping portion is given by

$$f_{\text{Gen.Gaussian}}(z;\infty,\tau) = 1/w, \tag{S1.1}$$

and its vertical intercept is 0.5, so we can define the CDF as follows:

$$P = F_{\text{Gen.Gaussian}}(z;\infty,\tau) = z/w + 0.5 \quad \text{for } -w/2 < z < w/2. \tag{S1.2}$$

The z-value corresponding to the threshold, P_θ, is given by inverting Equation (S1.2):

$$\begin{aligned} F^{-1}_{\text{Gen.Gaussian}}(P_\theta;\infty,\tau) &= w(P_\theta - 0.5) \\ &= \frac{w(1-1/e)}{2}. \end{aligned} \tag{S1.3}$$

We can use the expression for $F^{-1}_{\text{Gen.Gaussian}}(P_\theta;\infty,\tau)$ in Equation (S1.3) to substitute for $F^{-1}(P_\theta)$ in Equation (16) of the main paper, and we can use the expression for $f_{\text{Gen.Gaussian}}(\cdot;\infty,\tau)$ in Equation (S1.1) to substitute for $f(\cdot)$ in Equation (16). This gives us

$$\beta^{\text{Noise}}_{\text{Gen.Gaussian}}(\infty) = e - 1. \ \square$$
