# Supplementary material for: Four Theorems on the Psychometric Function
Source: PLoS One. 2013 Oct 4;8(10):e74815. doi: 10.1371/journal.pone.0074815 (PMC3790801; doi:10.1371/journal.pone.0074815)
Supplement: Appendix S2 — Point of inflection of the Legge-Foley transducer. (PDF) [file pone.0074815.s002.pdf]

Appendix S2. Point of inflection of the Legge-Foley transducer

The Legge-Foley transducer is given by

$$R(x) = \frac{x^u}{k^v + x^v} . \quad \text{(S2.1)}$$

We can ignore any vertical scaling because it does not affect the position of the point of inflection along the horizontal axis. The point of inflection is the peak of the gradient, and can be found by finding the input level at which the second derivative, $R''(x)$, is zero.

We start by finding the first derivative:

$$R'(x) = \frac{uk^v x^{u-1} + (u-v)x^{u+v-1}}{\left(k^v + x^v\right)^2} . \quad \text{(S2.2)}$$

Then the second derivative is given by

$$R''(x) = \left\{ \left(k^v + x^v\right)\left[u(u-1)k^v x^{u-2} + (u-v)(u+v-1)x^{u+v-2}\right] - 2vx^{v-1}\left[uk^v x^{u-1} + (u-v)x^{u+v-1}\right] \right\} \div \left(k^v + x^v\right)^3 . \quad \text{(S2.3)}$$

The point of inflection occurs when $R''(x) = 0$, and, setting $R''(x)$ to 0 in Equation (S2.3) gives a quadratic equation in x^v:

$$A\left(x^v\right)^2 + Bk^v x^v + Ck^{2v} = 0 , \quad \text{(S2.4)}$$

where

$$A = (u-v)(u-v-1) , \quad \text{(S2.5)}$$
$$B = (u-v)(u+v-1) + u(u-1) - 2uv , \quad \text{(S2.6)}$$
$$C = u(u-1) . \quad \text{(S2.7)}$$

We can therefore solve for x^v using the quadratic formula, which gives the following expression for x:

$$x = k\left(\frac{-B \pm \sqrt{B^2 - 4AC}}{2A}\right)^{1/v}. \tag{S2.8}$$

Equation (S2.8) shows that there are two values of x that satisfy Equation (S2.4). One of these will be the point of inflection of the Legge-Foley transducer, and the other will not. We now prove that the positive square root in (S2.8) yields a complex (i.e., non-real) value of x; since the position of the point of inflection along the abscissa must be a real number, this value of x cannot be the point of inflection, and so we need to take the negative square root instead. The explanation of why the positive square root gives a non-real solution runs as follows. Firstly, for all $v > 1$, and most $v < 1$, x will only be real if the expression inside the brackets is positive; this is because, for all $v > 1$, and most $v < 1$, $1/v$ is not an integer, and a negative number raised to a non-integer power is a complex number with a non-zero imaginary component. The sign of the expression inside the brackets depends on the signs of A, B, and C. For the Legge-Foley transducer to have a sigmoid shape, we need $u > 1$, so that it is expansive for low inputs, and we need $(u - v) < 1$ so that it is compressive for high inputs. $u > 1$ implies that $C > 0$, and $(u - v) < 1$ implies that A < 0. Therefore, from the signs of A and C, we have

$$\begin{aligned} & B^2 - 4AC > B^2 \\ \Rightarrow\quad & +\sqrt{B^2 - 4AC} > B \\ \Rightarrow\quad & -B + \sqrt{B^2 - 4AC} > 0 \\ \Rightarrow\quad & \frac{-B + \sqrt{B^2 - 4AC}}{2A} < 0 \end{aligned}$$

Thus, the positive square root in Equation (S2.8) will not yield a real value of x, so this solution cannot be the point of inflection, and we need to take the negative square root. At the point of inflection, then, we have

$$x = k\left(\frac{-B - \sqrt{B^2 - 4AC}}{2A}\right)^{1/v}. \square \tag{S2.9}$$

For Legge and Foley's parameters ($u = 2.4$; $v = 2$), the point of inflection occurs at $x = 0.816k$. For Meese, Georgeson and Baker's parameters ($u = 3.53$; $v = 3.07$), the point of inflection occurs at $x = 0.952k$.
